# Ketogenic diet-produced β-hydroxybutyric acid accumulates brain GABA and increases GABA/glutamate ratio to inhibit epilepsy

**DOI:** 10.1038/s41421-023-00636-x

**Published:** 2024-02-13

**Authors:** Ya-Nan Qiao, Lei Li, Song-Hua Hu, Yuan-Xin Yang, Zhen-Zhen Ma, Lin Huang, Yan-Peng An, Yi-Yuan Yuan, Yan Lin, Wei Xu, Yao Li, Peng-Cheng Lin, Jing Cao, Jian-Yuan Zhao, Shi-Min Zhao

**Affiliations:** 1 The Obstetrics & Gynaecology Hospital of Fudan University, State Key Laboratory of Genetic Engineering, Shanghai Key Laboratory of Metabolic Remodelling and Health, Institutes of Biomedical Sciences, and Children’s Hospital of Fudan University, Fudan University, Shanghai, China; 2 Department of Anatomy, School of Basic Medical Sciences, Zhengzhou University, Zhengzhou, Henan China; 3 Department of Cell Biology, Blavatnik Institute, Harvard Medical School, Boston, MA USA; 4 Key Laboratory for Tibet Plateau Phytochemistry of Qinghai Province, College of Pharmacy, Qinghai University for Nationalities, Xining, Qinghai China; 5 Institute for Developmental and Regenerative Cardiovascular Medicine, MOE-Shanghai Key Laboratory of Children’s Environmental Health, Xinhua Hospital, Shanghai Jiao Tong University School of Medicine, Shanghai, China

**Keywords:** Post-translational modifications, Cancer metabolism, Mechanisms of disease

## Abstract

Ketogenic diet (KD) alleviates refractory epilepsy and reduces seizures in children. However, the metabolic/cell biologic mechanisms by which the KD exerts its antiepileptic efficacy remain elusive. Herein, we report that KD-produced β-hydroxybutyric acid (BHB) augments brain gamma-aminobutyric acid (GABA) and the GABA/glutamate ratio to inhibit epilepsy. The KD ameliorated pentetrazol-induced epilepsy in mice. Mechanistically, KD-produced BHB, but not other ketone bodies, inhibited HDAC1/HDAC2, increased H3K27 acetylation, and transcriptionally upregulated *SIRT4* and glutamate decarboxylase 1 (*GAD1*). BHB-induced SIRT4 de-carbamylated and inactivated glutamate dehydrogenase to preserve glutamate for GABA synthesis, and GAD1 upregulation increased mouse brain GABA/glutamate ratio to inhibit neuron excitation. BHB administration in mice inhibited epilepsy induced by pentetrazol. BHB-mediated relief of epilepsy required high GABA level and GABA/glutamate ratio. These results identified BHB as the major antiepileptic metabolite of the KD and suggested that BHB may serve as an alternative and less toxic antiepileptic agent than KD.

## Introduction

The ketogenic diet (KD) switches cell energy metabolism from carbohydrates to fatty acid and amino acid oxidation^1^, from which ketone bodies, such as β-hydroxybutyrate (BHB), acetone, and acetoacetate (AcAc), are generated^2^. A consensus is yet to be reached regarding whether physiologic effects of the KD, including weight loss^3^, decreasing blood sugar via normalizing beta cell function^4^, and cholesterol level reduction^5^, are due to altered energy metabolism or ketone body production^6,7^. One of the recognized efficacies of the KD is its beneficial neuronal effects. The KD has been reported to help treat brain disorders such as Parkinson’s disease^8^, Alzheimer’s disease^9^, multiple sclerosis^10,11^, sleep disorders^12^, autism^13^, and brain cancer^14^. Since its first clinical practice in epilepsy treatment in 1921^15^, KD has been shown to be effective in treating children with refractory epilepsy^16,17^, in which onset often occurs in childhood and older adulthood^18^.

Epilepsy is a common neurological condition which affects 0.8%–1% of the human population, and ~30% of patients with epilepsy have drug-resistant epilepsy. Epileptic seizures are mostly due to a relative imbalance of excitatory and inhibitory neurotransmitters, including glutamate and gamma-aminobutyric acid (GABA)^19^, that leads to abnormal and excessively synchronized firing of neurons in the brain^20^. In the central nervous system, GABA is primarily produced by glutamate decarboxylase 1 (GAD1), which decarboxylates glutamate within GABAergic axon terminals^21^. The interictal epileptic activity in the human temporal lobe is critically dependent on the depolarizing action of GABA^22^. Disturbance of central glutamate and GABA homeostasis is consistently associated with neurological disorders, such as schizophrenia and epilepsy^23,24^. The proposed antiepileptic mechanisms include inhibiting the presynaptic release of glutamate by directly competing with Cl^–^ for allosteric activation of vesicular glutamate transporters and increasing levels of GABA in cerebrospinal fluid^25^. BHB can be produced in astrocytes from oxidation of fatty acids or catabolism of amino acids, and is metabolized in the mitochondria of all brain cell types^26^. The open probability of K_ATP_ channels in the hippocampus in vitro is enhanced in the presence of BHB^27^.

Glutamate receives amines from most amino acids for deamination and is the most abundant excitatory neurotransmitter in the vertebrate nervous system^28^. Glutamate can be converted to α-ketoglutarate (α-KG) to enter the tricarboxylic acid cycle^29^ via glutamate dehydrogenase (GDH) or to GABA via GAD1. It has been suggested that SIRT4 represses enzymatic activity and limits the catabolism of glutamate and glutamine by ADP-ribosylation of GDH^30^. GDH-produced ammonia is converted to carbamoyl phosphate (CP), which spontaneously forms protein lysine carbamylation (CP-K) to activate mitochondrial proteins, including ornithine transcarbamylase (OTC) of the urea cycle; CP-K effects on OTC are reversed via mitochondrial sirtuin SIRT4^31^. SIRT4 regulates GDH activity and GABA/glutamate ratio that determines neuronal excitation, it is possible that CP-K and SIRT4 can regulate glutamate and GABA homeostasis, and consequently determine GABA/glutamate ratio in epilepsy control.

## Results

### KD increased the GABA, glutamate, and GABA/glutamate ratio in the brains of mice

**Fig. 1:**
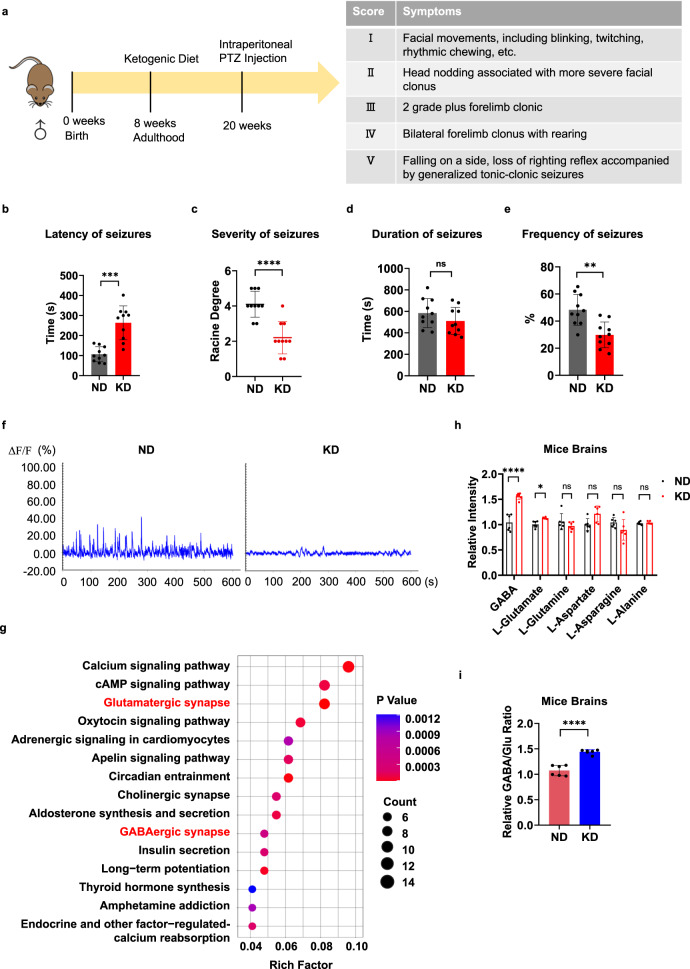
KD increased GABA, glutamate, and GABA/glutamate ratio in mouse brains. **a** Schematic diagram of the timeline of dietary exposure and PTZ treatment of mice and the Racine scale used to assess the intensity of epilepsy in mice. **b**–**e** PTZ-induced epilepsy in mice. KD- and ND-fed mice were intraperitoneally administered with a shot of PTZ before being monitored for epilepsy symptoms that were staged by the Racine scale. Latency time (**b**), the highest level (**c**), duration of (**d**) and frequency of seizures (**e**) were monitored. Data are means ± SD, *n* = 10 mice in each treatment. **f** The KD weakened neuronal activity in mice. The representative EEG recordings of WT mice fed the ND or KD with epileptiform activity are shown. **g** Pathway enrichment analysis showing metabolic pathways affected by the KD. The rich factors represent the ratio of differentially expressed protein in this pathway annotated by number. **h** The KD increased GABA and glutamate in mouse brains. Levels of GABA, glutamate, glutamine and aspartate were determined in the brains of C57 mice fed with either ND or KD. *n* = 6 mice. **i** The KD increased the GABA/glutamate ratio in mouse brains. The ratio of GABA/glutamate was compared between ND- and KD-fed mice. *n* = 6 mice.

To investigate the seizure-preventing effects of the KD, 8-week-old male C57/BL6 mice were fed with either a KD or normal diet (ND) for 12 weeks before they were intraperitoneally injected with pentetrazol (PTZ), which induces acute epilepsy in mice^32^. Female mice were not used in this study to exclude the effects of hormone changes during puberty and hormone fluctuations over estrous cycle^33^. The development of both clonic and tonic-clonic seizures began with stereotypic behaviors, such as Straub tail, myoclonic jerks and hindlimb flexions, which progressed to forelimb clonus and head bobbing and ended with relaxed muscle tone. And an isoelectric period^34^ was monitored. The severity of epilepsy was calculated according to the Racine scale (Fig. 1a)^35^.

The KD-fed mice had a longer seizure latency than the ND-fed mice (Fig. 1b). Moreover, the KD-fed mice also had less severe seizure symptoms. Among ten mice tested in each group, only three KD-fed mice reached grade III or above; in contrast, most ND-fed mice reached grade IV or V (Fig. 1c). Furthermore, while PTZ induced a similar epilepsy duration in both KD- and ND-fed mice (Fig. 1d), it induced lower seizure frequency in KD-fed mice than in ND-fed mice (Fig. 1e). The epilepsy-relieving effects of the KD were further confirmed via electroencephalography (EEG), which showed that PTZ-induced less electrical activity in KD-fed mice than in ND-fed mice (Fig. 1f).

Proteomic analysis revealed that metabolic pathways, including glutamatergic and GABA-like receptor-related pathways, were altered by the KD (Fig. 1g). These results were consistent with those of the targeted metabolomic analyses, which showed that KD-fed mice had higher levels of glutamate, GABA, and associated metabolites in the brains than those of ND-fed mice (Fig. 1h). Notably, KD feeding resulted in a higher GABA/glutamate ratio (Fig. 1i). These findings highlighted the possibility that the KD exerts seizure-relieving effects via augmenting GABA and GABA/glutamate ratio, both inhibit the excitation of neurons^36^.

### KD inactivated GDH and activated GAD1

**Fig. 2:**
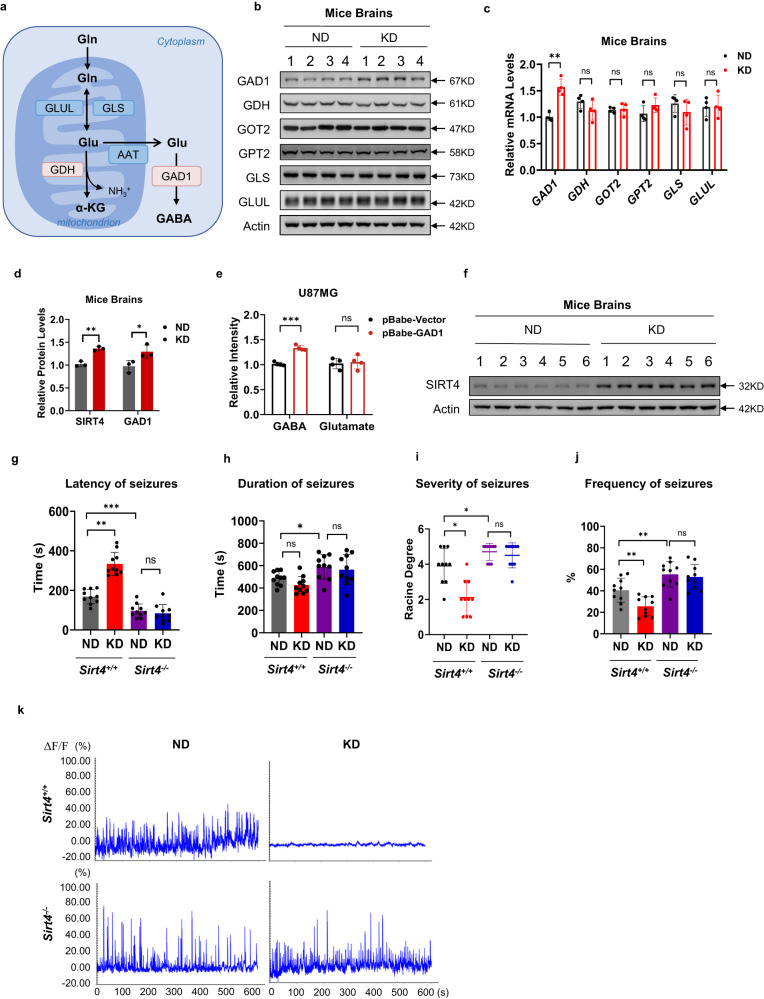
KD relieved epilepsy by changing GDH, GAD1, and SIRT4. **a** Diagram of the enzymes involved in glutamate/GABA metabolism pathways. Enzymes studied in the current study (GDH and GAD1) are highlighted. **b**, **c** The KD upregulated GAD1 expression. Protein (**b**) and mRNA (**c**) levels of glutamate/GABA metabolism enzymes were determined in the brains of mice fed ND or KD. *n* = 4 mice. **d** KD induced SIRT4 and GAD1 expression. The histogram reflects the protein levels of SIRT4 and GAD1. *n* = 3 mice. **e** GAD1 had a limited impact on glutamate levels. GAD1 was overexpressed in U87MG cells; GABA and glutamate levels were compared between overexpressed and control U87MG cells. **f** The KD induced SIRT4 expression. SIRT4 levels in mouse brains were compared between KD- and ND-fed mice. *n* = 4 mice. **g**–**k** SIRT4 was required for the KD to relieve epilepsy in mice. KD- and ND-fed WT and *Sirt4*^*–/–*^ mice were each administered with an intraperitoneal PTZ injection before being monitored for epilepsy symptoms. Latency time (**g**), duration (**h**), the highest level (**i**), and frequency of seizures (**j**) and EEG (**k**) were monitored. Data were means ± SD, *n* = 10 mice in each treatment.

The KD altered glutamate homeostasis, prompting us to examine whether it affected the levels of enzymes involved in the Glu/GABA metabolism pathway (Fig. 2a) in vivo^37^. The KD had negligible impacts on other Glu/GABA metabolism pathway enzymes, except for increased mRNA and protein levels of GAD1 (Fig. 2b, c), consistent with the proteomics results (Figs. 1g, 2d; Supplementary Fig. S1a). As the KD caused neither GDH nor glutaminase (GLS) changes in mouse brains (Fig. 2b, c), it may increase glutamate (see Fig. 1h) by inactivating GDH, which oxidizes glutamate to α-KG, or by inactivating GAD1, which converts glutamate to GABA^38^. When GAD1 was overexpressed in U87MG (a human brain glioblastoma cell line) cells, it increased GABA levels; however, it failed to decrease glutamate levels (Fig. 2e), suggesting that GAD1 activity is more important for GABA/glutamate ratio than for glutamate levels. Collectively, these results suggest that the KD may decrease GDH-specific activity to increase glutamate in mouse brains.

### SIRT4 was involved in seizure relief via the KD

Notably, SIRT4, a known GDH regulator^30^, was also induced by the KD (Fig. 2d; Supplementary Fig. S1a). Upregulation of SIRT4 by the KD was confirmed via western blot (Fig. 2f) and real-time qPCR (Supplementary Fig. S1b) in the mice brains. The involvement of SIRT4 in the KD-mediated control of epilepsy was further investigated. 8-week-old wild-type (WT) and *Sirt4* knockout male C57 mice (*Sirt4*^–*/*–^ mice) were fed with ND or KD. The *Sirt4*^*–/–*^ mice grew normally, similar to WT mice, upon chow feeding (Supplementary Fig. S1c, d). Moreover, indiscernible differences in the brain tissue structure were observed using Nissl (Supplementary Fig. S1e) and hematoxylin and eosin (HE) staining (Supplementary Fig. S1f) after 12 weeks of ingesting KD. PTZ-induced pronounced epilepsy symptoms in *Sirt4*^*–/–*^ mice, as manifested by faster onset of symptoms such as limb tremors and side-to-side swaying (Fig. 2g), accompanied by longer seizures (Fig. 2h). *Sirt4*^*–/–*^ mice exhibited more frequent and intense symptoms, including outstretched hind limbs and front paws tucked under the chin, which suggest death during an episode of status epilepticus (Fig. 2i, j) upon PTZ administration. These behavioral seizures and epileptiform interictal activity in PTZ-challenged *Sirt4*^*–/–*^ mice were confirmed using video/EEG (Fig. 2k). Notably, the KD failed to relieve the epilepsy symptoms in PTZ-challenged *Sirt4*^*–/–*^ mice (Fig. 2g, k). Collectively, these results suggest that the KD prevents epilepsy, which is dependent on SIRT4 function.

We injected Cre-adeno-associated virus (AAV) into the hippocampus of *Sirt4*^*flox/flox*^ mice to delete *Sirt4* from hippocampus (*Sirt4-cKO*, Supplementary Fig. S2a, b). We found that *Sirt4-cKO* mice phenocopied constitutive *Sirt4* knockout mice in response to PTZ challenge and to KD feeding (Supplementary Fig. S2c–i). These results confirmed that *Sirt4* in central nervous system is involved in seizure relief effects of the KD.

### KD-produced BHB-activated SIRT4 and GAD1 transcription

We investigated how the KD induces *SIRT4* and *GAD1* expression. The KD has a low carbohydrate content. Therefore, we tested whether sugar deprivation could result in KD-induced *SIRT4* expression. Culturing U87MG (Supplementary Fig. S3a) and mouse neuroblastoma (Neuro-2a) (Supplementary Fig. S3b) cells in low-glucose medium failed to induce changes in *SIRT4* and *GAD1* protein and mRNA levels, ruling out the possibility that the KD drives *SIRT4* overexpression solely owing to its low carbohydrate content.

**Fig. 3:**
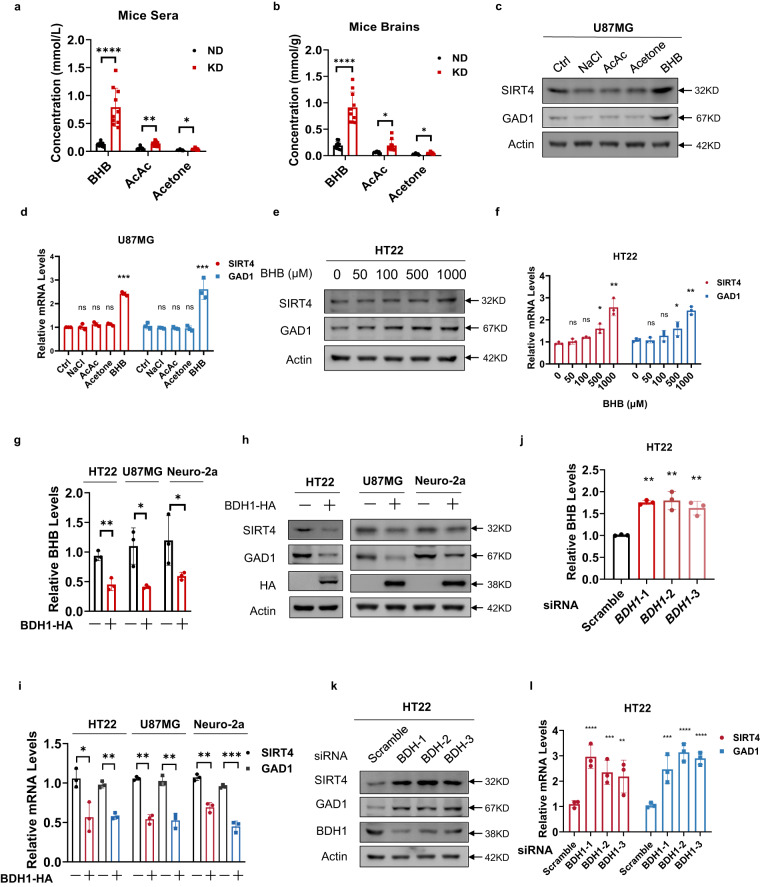
KD-produced BHB activated *SIRT4* and *GAD1* transcription. **a**, **b** The KD increased mice ketone body levels. Serum (**a**) and brain (**b**) levels of the BHB, AcAc, and acetone were detected in ND- and KD-fed mice. **c**, **d** BHB increased SIRT4 and GAD1 expression levels. U87MG cells were treated with ketone bodies and the protein (**c**) and mRNA (**d**) levels of SIRT4 and GAD1 were measured. **e**, **f** BHB induced SIRT4 and GAD1 expression in a dose-dependent manner. Protein (**e**) and mRNA (**f**) levels of SIRT4 and GAD1 were determined in HT22 cells that were treated with BHB at the indicated concentrations. **g** *BDH1* overexpression decreased BHB levels. The BHB levels were measured in HT22, U87MG and Neuro-2a cells and in *BDH1*-overexpressing HT22 cells, U87MG and Neuro-2a cells. **h**, **i** *BDH1* overexpression decreased SIRT4 and GAD1 expression. Protein (**h**) and mRNA (**i**) levels of SIRT4 and GAD1 were detected in HT22 cells, U87MG, and Neuro-2a cells and in *BDH1*-overexpressing HT22 cells, U87MG and Neuro-2a cells. **j**–**l** *BDH1* knockdown increased BHB levels and increased SIRT4 and GAD1. *BDH1* was silenced with independent siRNAs in HT22 and U87MG cells. BHB levels (**j**), protein (**k**), and mRNA(**l**) levels of SIRT4 and GAD1 were compared between siRNA-untreated and -treated cells.

Ketone bodies, such as BHB, AcAc, and acetone, significantly (*P* < 0.05) increased in the serum (Fig. 3a) and brain (Fig. 3b) of KD-fed mice. However, at physiologic relevant higher levels, only BHB-induced protein and mRNA expression of *SIRT4* and *GAD1*, when U87MG and Neuro-2a cells were treated with each of 5 mM sodium BHB, sodium AcAc, or sodium chloride, or with 0.5 mM acetone (Fig. 3c, d; Supplementary Fig. S3c). Moreover, the BHB-induced protein and mRNA expression of *SIRT4* and *GAD1* was dose-dependent in mouse hippocampal HT22 cells, and in U87MG (Fig. 3e, f; Supplementary Fig. S3d). To further determine the effect of BHB on *SIRT4* and *GAD1* expression, we overexpressed β-hydroxybutyrate dehydrogenase 1 (*BDH1*), which catalyzes the first reaction of BHB oxidation to convert BHB back into AcAc^39^, in HT22, U87MG, and Neuro-2a cells. We found *BDH1* overexpression, while decreasing cellular BHB levels (Fig. 3g), decreased the expression of *SIRT4* and *GAD1* in HT22, U87MG, and Neuro-2a cells (Fig. 3h, i). Conversely, knockdown *BDH1* with small interfering RNAs that increased intracellular BHB concentration (Fig. 3j), increased mRNA and protein levels of *SIRT4* and *GAD1* in U87MG and HT22 cells (Fig. 3k, l; Supplementary Fig. S3f). These results, together with the fact that *SIRT4* and *GAD1* degradation in protein synthesis-inhibited CHX-treated cell lysates were unaffected by BHB (Supplementary Fig. S3g), supported the hypothesis that the KD promotes *SIRT4* and *GAD1* transcription by upregulating BHB levels.

### BHB inhibited HDAC1/HDAC2, thus activating *SIRT4* and *GAD1* transcription

**Fig. 4:**
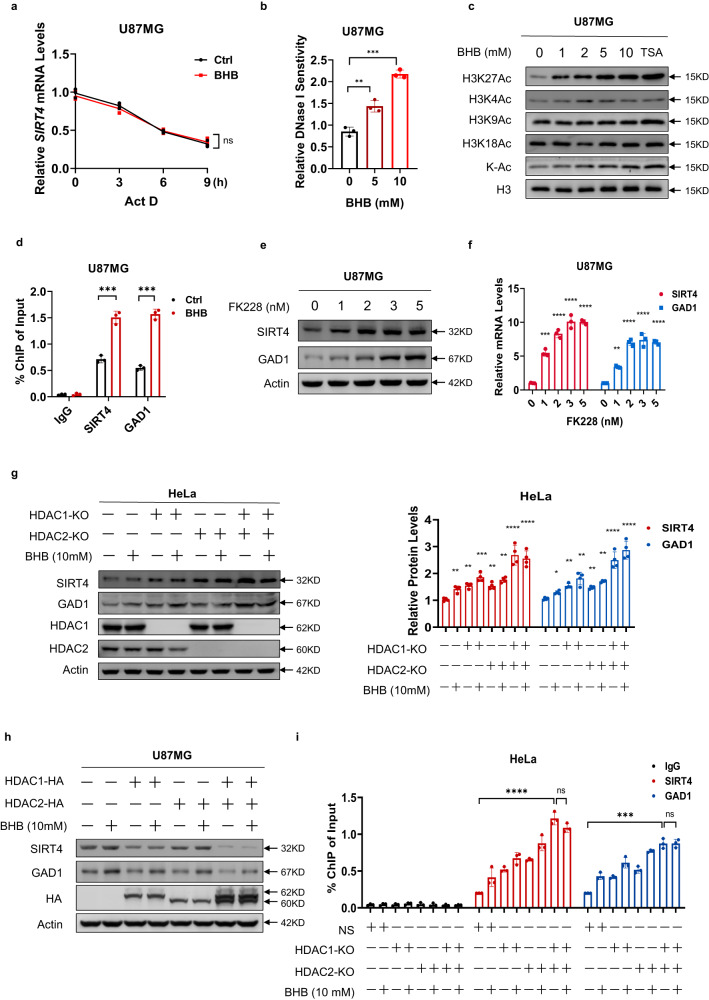
BHB inhibited HDAC1/HDAC2 to activate *SIRT4* and *GAD1* transcription. **a** mRNA degradation did not account for the BHB-promoted *SIRT4* and *GAD1* mRNA increase. *SIRT4* mRNA in U87MG cells was detected at the indicated time points with 10 mM actinomycin D. **b** BHB increased chromatin accessibility of the *SIRT4* promoter. Quantitative PCR was performed on DNase I-pretreated nuclei of U87MG cells that were treated with BHB with the indicated concentrations. **c** BHB specifically elevated H3K27Ac. Histone acetylation levels were assayed in U87MG cells and in BHB- or trichostatin A (TSA)-treated U87MG cells, employing site-specific and pan-anti-acetyl-lysine (Ac) antibodies. **d** BHB-treatment elevated H3K27Ac at the promoter regions of *SIRT4* and *GAD1*. The H3K27Ac antibody was used for immunoprecipitation (IP) after BHB treatment, and qRT-PCR of *SIRT4* and *GAD1* promoter output was performed. **e**, **f** The HDAC1/HDAC2-specific inhibitor FK228 increased SIRT4 and GAD1 protein (**e**) and mRNA (**f**) levels. The ability of FK228 to upregulate SIRT4 and GAD1 expression was tested in U87MG cells. **g**, **h** BHB elevated SIRT4 and GAD1 expression, dependent on HDAC1 and HDAC2 acetylation. The effects of BHB (10 mM) treatment on SIRT4 and GAD1 expression were assayed in HeLa cells with *HDAC1*, *HDAC2*, or double knockout of both (**g**, quantified 4 replicates in right), and in U87MG cells overexpressing *HDAC1*, *HDAC2*, or both (**h**). **i** *HDAC1* and *HDAC2* knockout enhanced H3K27Ac at the *SIRT4* promoter. ChIP assays were performed for the promoter region of *SIRT4*, using the H3K27Ac antibody for IP and qRT-PCR for the *SIRT4* promoter output.

To investigate the mechanism by which BHB promotes *SIRT4* and *GAD1* expression in cells, we used actinomycin D to inhibit mRNA synthesis and measured the half-lives of *SIRT4* and *GAD1* mRNA in U87MG cells. No significant differences in *SIRT4* and *GAD1* mRNA half-lives were observed between the BHB-treated and control cells (Fig. 4a; Supplementary Fig. S4a), indicating that BHB promotes *SIRT4* and *GAD1* mRNA synthesis rather than inhibiting mRNA degradation. Given that BHB is an established inhibitor of histone deacetylases (HDACs)^40^, we analyzed the chromatin accessibility of the *SIRT4* and *GAD1* promoter regions and found that BHB treatment markedly enhanced chromatin accessibility of the *SIRT4* and *GAD1* promoters (Fig. 4b; Supplementary Fig. S4b). This result is consistent with the key function of BHB, which enables the transcriptional machinery to inhibit histone deacetylation^40^.

Tracing the acetylation levels at different sites on histone H3 via immunoblotting revealed that the acetylation levels at H3K27 increased with BHB (Fig. 4c). Moreover, chromatin immunoprecipitation (ChIP) assays showed that BHB enhanced H3K27Ac levels and histone interactions with *SIRT4* and *GAD1* promoters (Fig. 4d), suggesting that BHB-induced *SIRT4* and *GAD1* transcriptional activation may correlate with H3K27Ac, which is deacetylated by the HDAC family, including HDAC1–HDAC10^41^.

Treatment of U87MG cells with different HDAC inhibitors revealed that neither the HDAC3 inhibitor RGFP966 (Supplementary Fig. S4c, d) nor the HDAC 4/5/7/9 inhibitor TMP195 (Supplementary Fig. S4e, f) affected the expression of *SIRT4* and *GAD1*, and only the HDAC1 and HDAC2 inhibitor FK228 upregulated protein (Fig. 4e) and mRNA (Fig. 4f) levels of *SIRT4* and *GAD1*.

To determine whether HDAC1 and HDAC2 are involved in the transcriptional regulation of *SIRT4* and *GAD1* via BHB, we knocked out either *HDAC1* or *HDAC2* to induce *SIRT4* and *GAD1* transcription and protein expression; however, only knockout of both *HDAC1* and *HDAC2* caused pronounced increase of *SIRT4* and *GAD1* expression and prevented BHB from activating *SIRT4* and *GAD1* transcription in HeLa cells (Fig. 4g). In contrast, the overexpression of *HDAC1* and *HDAC2* in U87MG (Fig. 4h) and HeLa cells (Supplementary Fig. S4g) decreased *SIRT4* and *GAD1* expression. These results, consistent with those of ChIP assays, showed that *HDAC1* and *HDAC2* knockouts extensively increased the H3K27Ac of histones, which interacted with the *SIRT4* and *GAD1* promoters in HeLa cells (Fig. 4i). HDAC1/HDAC2-mediated histone deacetylation inhibits the transcription of *GAD1*^42^ and this study confirmed that BHB activates *SIRT4* and *GAD1* transcription by inhibiting HDAC1- and HDAC2-induced H3K27 deacetylation.

### Lysine carbamylation-activated GDH

**Fig. 5:**
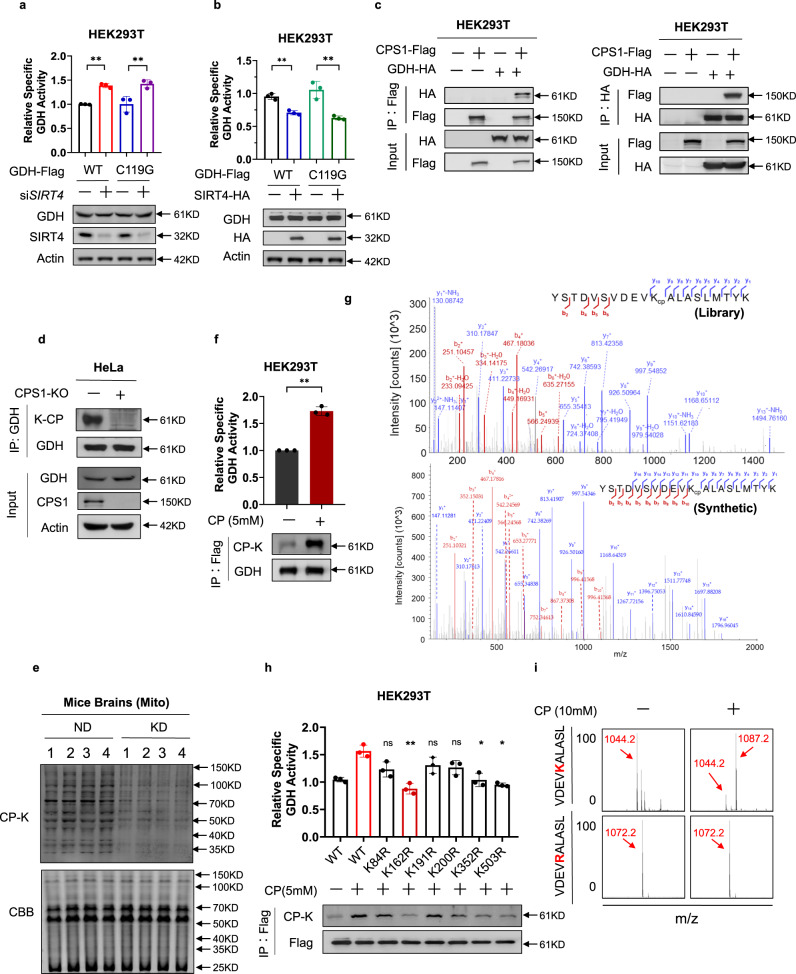
Lysine carbamylation activated GDH. **a**, **b** The activity of the ADP-ribosylation-null GDH^C119G^ mutant was under regulation by SIRT4. Flag-tagged GDH or GDH^C119G^ was ectopically expressed in HEK293T cells or *SIRT4*-silenced HEK293T cells (**a**). Flag-tagged GDH or GDH^C119G^ was ectopically expressed in HEK293T cells or *SIRT4*-overexpressing HEK293T cells (**b**). The specific activities of GDH and the GDH^C119G^ mutant were determined and compared. **c** GDH interacted with CPS1. Flag-tagged CPS1 or GDH was ectopically expressed in HEK293T cells together with individual HA-tagged GDH or CPS1 as indicated. Flag bead- or HA bead-purified proteins were subject to western blotting analysis. **d** Carbamylation of endogenous GDH relied on CPS1 to generate CP. Endogenous GDH of HeLa cells was purified via IP with Protein A beads conjugated with the GDH antibody, and carbamylation of the purified GDH was detected. **e** KD decreased mouse brain mitochondrial CP-K levels. Mitochondrial CP-K levels were detected in brains of mice fed with either ND or KD. **f** Carbamylation activated GDH. Flag-tagged GDH was ectopically expressed in HEK293T cells treated with CP (5 mM) and purified via IP with Flag beads. The specific activity of GDH was determined. **g** GDH K162 was carbamylated. MS/MS spectra from tryptic peptide libraries of HEK293T cells matching MS/MS spectra from synthetic peptide (composed of ^12^C, ^1^H, ^14^N, and ^16^O) identified GDH CP-K162. **h** Screening of the major regulatory carbamylation sites in GDH. Detected carbamylated residues were each mutated to arginine (R), and the specific activity of each mutant GDH was compared with that of WT GDH. **i** In vitro formation of CP-K162. The synthetic GDH K162-containing peptide was treated with 10 mM CP, followed by mass spectrometry analysis.

Although GAD1 activity was unresponsive to SIRT4 variation (Supplementary Fig. S5a, b) in *SIRT4*-silenced and *SIRT4*-overexpressing HEK293T cells, GDH was activated (Fig. 5a) and inactivated (Fig. 5b), respectively, suggesting that GDH is inactivated by GDH mono-ADP-ribosylation^43^. However, the activity of the ADP-ribosylation-null mutant GDH^C119G^ ^44^ was still responsive to SIRT4 variation (Fig. 5a, b), suggesting that a mechanism other than ADP-ribosylation regulates GDH enzyme activity to convert glutamate into α-KG.

Glutamate oxidation via GDH produces α-KG and ammonia, which are converted to CP that spontaneously forms CP-K^45,46^, which is reversed by SIRT4^31^. GDH interacted with CPS1, an enzyme that spontaneously produces CP to carbamylate lysine (Fig. 5c)^45^. Moreover, *CPS1* knockout in HeLa cells reduced the level of GDH CP-K (Fig. 5d). Furthermore, in the mitochondria of mouse brains, the KD-induced lower CP-K levels (Fig. 5e). Thus, we investigated the possibility that the KD induces SIRT4, which inactivates GDH by decarbamylating it. Moreover, the incubation of recombinant GDH with 5 mM CP increased GDH CP-K levels and activated GDH (Fig. 5f), suggesting that carbamylation activates GDH.

Using a Thermo Q-Exactive HF (QE) Quadrupole-Obitrap mass spectrometer, we detected multiple CP-modified lysine sites, including K84, K162, K191, K200, K352, and K503 (Fig. 5g; Supplementary Fig. S6) in GDH purified from U87MG cells. Eliminating potential carbamylation sites in GDH by switching lysine to arginine (K/R) revealed that GDH^K162R^, GDH^K352R^, and GDH^K503R^ inactivated GDH and diminished the ability of CP to activate GDH (Fig. 5h), suggesting that these were the key carbamylation sites that activated GDH. To confirm that CP spontaneously carbamylates GDH, we synthesized a GDH peptide containing K162, which is evolutionarily conserved from *Trachemys scripta elegans* to *Homo sapiens* (Supplementary Fig. S7a), and close to the active site of GDH, K183 (Supplementary Fig. S7b). Treatment with 5 mM CP induced carbamylation in the K162-containing peptide but not in the peptide in which K162 was replaced by arginine (K162/R) (Fig. 5i). Collectively, these results suggest that lysine carbamylation enhances GDH activity without affecting its expression.

### SIRT4 decarbamylated and inactivated GDH to increase both glutamate and GABA but GABA/glutamate ratio

**Fig. 6:**
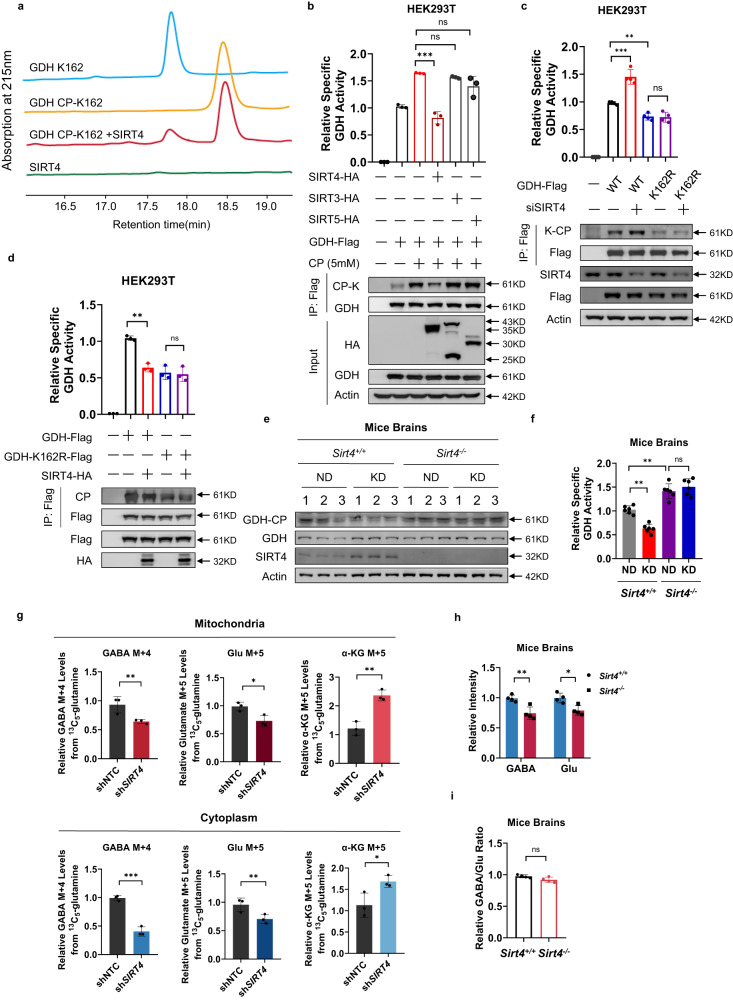
SIRT4 decarbamylated and inactivated GDH to increase glutamate and GABA and not the GABA/glutamate ratio. **a** SIRT4 decarbamylated GDH CP-K162. RP-HPLC analyses were used to detect decarbamylation of the synthetic GDH CP-K162 peptide catalyzed by SIRT4. **b** SIRT4 decarbamylated GDH. HA-tagged mitochondrial SIRT3, SIRT4, and SIRT5 were each individually expressed in untreated and CP (5 mM)-treated HEK293T cells. The GDH ectopically expressed in these cells were purified and subject to CP-K and specific activity analysis. **c**, **d** K162 was the major regulatory carbamylation site in GDH. Ectopically expressed GDH and GDH^K162R^ mutant from HEK293T cells and *SIRT4* knockdown (**c**) or *SIRT4* overexpression (**d**) HEK293T cells assayed for CP-K and specific activities. **e** GDH in *Sirt4*^*–/–*^ mouse brain was more carbamylated. The GDH protein was purified using Protein A beads conjugated with the GDH antibody from the brains of WT and *Sirt4*^*–/–*^ mice that were fed either ND or KD. GDH carbamylation was detected for purified GDH. **f** GDH was inactivated by the KD only in WT mouse brains. The WT and *Sirt4*^*–/–*^ mice were each fed the ND and KD from week 8. The mouse brain GDH purified at week 20 was assayed for specific activity. **g** *SIRT4* knockdown decreased GABA and glutamate and increased α-KG. Culture media was supplemented with 2 mM ^13^C-glutamine. The M + 5 glutamate and α-KG (carbons fully labeled) and M + 4 GABA (carbons fully labeled) were determined in the mitochondria and cytoplasm of WT and *SIRT4* knockdown U87MG cells after 6 h of chasing. **h** *Sirt4* ablation caused more pronounced GABA elevation than glutamate elevation. The levels of GABA and glutamate were compared between WT and *Sirt4*^*–/–*^ mouse brains. **i** *Sirt4* ablation did not alter GABA/glutamate ratio in mouse brains. GABA/glutamate ratios were detected in WT and *Sirt4*^*–/–*^ mouse brains.

GDH and SIRT4 were co-immunoprecipitated when they were simultaneously expressed in HEK293T cells (Supplementary Fig. S8a). Moreover, the decarbamylase activity of SIRT4 was confirmed by detecting decarbamylated peptide formation from the K162R-containing GDH peptide using reverse-phase high-performance liquid chromatography (Fig. 6a). This suggests that SIRT4 is a decarbamylase of GDH, which was confirmed by the CP-K level and specific activity of ectopically expressed GDH in HEK293T cells (Fig. 6b). Endogenous GDH in U87MG cells (Supplementary Fig. S8b) cultured in CP-supplemented media was decreased by *SIRT4* overexpression, and not by that of SIRT3 or SIRT5. Conversely, silencing *SIRT4* in HEK293T cells increased CP-K levels and the specific activity of GDH (Fig. 6c). In contrast, SIRT4 had no effect on the specific activity of GDH^K162R^, which was diminished when it was co-expressed with GDH^K162R^ (Fig. 6c, d). Affinity-purified GDH from WT mice brains were carbamylated less and were less active than that from *Sirt4*^*–/–*^ mouse brains (Fig. 6e, f). Moreover, the KD, which upregulated SIRT4 in the mouse brains (Fig. 2d–f), decreased CP-K levels and inactivated endogenous GDH (Fig. 6e, f). By using ^13^C-glutamine as a tracer (Supplementary Fig. S8c), we detected the relative cytosolic and mitochondrial ^13^C-GABA and ^13^C-α-KG levels in both U87MG cells and *SIRT4*-silenced U87MG cells. *SIRT4* knockdown decreased the GABA and glutamate levels, but increased α-KG levels (Fig. 6g). Moreover, the GABA and glutamate levels were decreased in *Sirt4*^*–/–*^ mouse brains (Fig. 6h), and the GABA/glutamate ratio was not altered by SIRT4 removal (Fig. 6i). These results suggest that GAD1 is not the downstream of SIRT4, and collectively supports the idea that SIRT4 decarbamylates and inactivates GDH.

### BHB effectively relieved epilepsy

To determine whether BHB alone may have seizure-relieving effects, the ND was supplemented with a BHB ester via oral gavage, which increased serum BHB levels > 4-fold (Supplementary Fig. S9a); however, it did not affect body weight (Supplementary Fig. S9b) and food intake (Supplementary Fig. S9c).

**Fig. 7:**
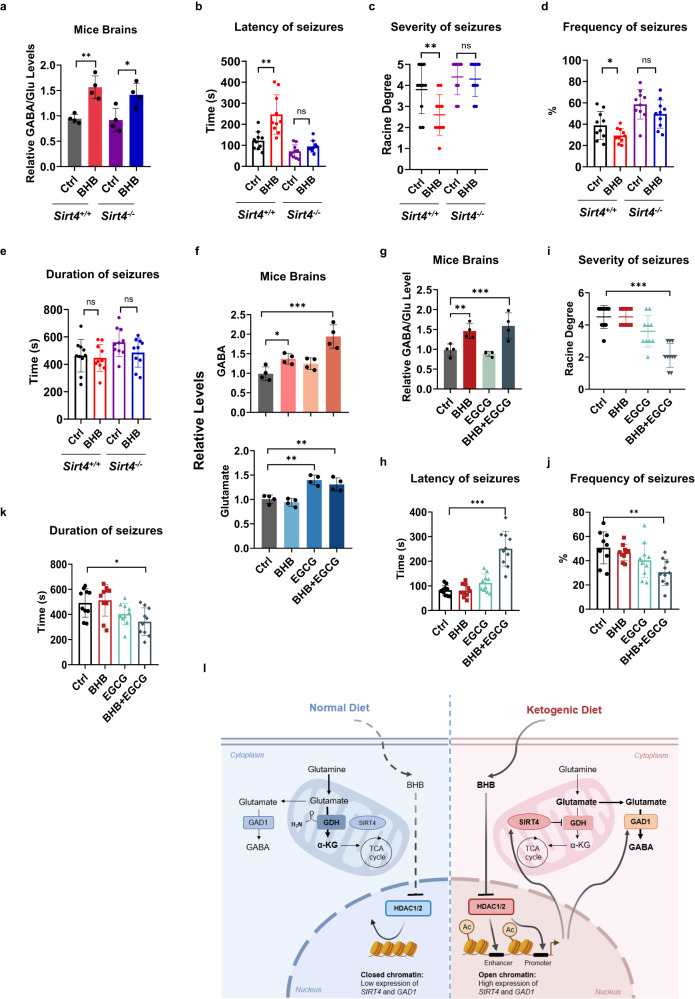
BHB relieved epilepsy in mice. **a** BHB increased the GABA/glutamate ratio in WT and *Sirt4*^*–/–*^ mouse brains. Data were represented as individual values. **b**–**e** BHB alone SIRT4-dependently relieved seizure phenotypes. The ND- and BHB-fed WT and *Sirt4*^*–/–*^ mice were subject to measurements after they were administered with PTZ. Latency time (**b**), the highest level (**c**), the frequency (**d**) and the duration of seizures (**e**) were monitored. Data were means ± SD, *n* = 10 mice in each treatment. **f**–**k** GDH inhibition potentiated BHB to relieve seizures. BHB or the GDH inhibitor EGCG alone, or BHB together with EGCG were employed to treat *Sirt4*^*–/–*^ mice. The seizure-relieving effects of these treatments were monitored after mice were administered with PTZ. Brain GABA and glutamate (**f**) and GABA/glutamate ratio (**g**), latency time (**h**), the highest level of seizures (**i**), the frequency of seizures (**j**) and the duration of seizures (**k**) were monitored. Data were means ± SD, *n* = 10 mice in each treatment. **l** Schematic illustration showing KD upregulates brain GABA levels and GABA/glutamate ratio. Compared to ND, KD promotes BHB, which inhibits HDAC1/HDAC2 to upregulate *SIRT4* and *GAD1* transcription, results in inactivated GDH and activated GAD1, and consequently higher GABA and GABA/glutamate ratio.

BHB supplementation induced higher GABA/glutamate ratio in the brains (Fig. 7a), longer latency of seizures (Fig. 7b), less severe seizures (Fig. 7c), and lower frequency of them (Fig. 7d). However, it had no impact on seizure duration (Fig. 7e) in PTZ-induced C57 mice, reinstating the antiepileptic efficacy of the KD (see Fig. 1b–e). Notably, no seizure-relieving efficacy was observed in PTZ-treated *Sirt4*^*–/–*^ mice (Fig. 7b, e). The lower potency of BHB in inhibiting epilepsy in *Sirt4*^*–/–*^ mice than in WT mice was further confirmed by video/EEG (Supplementary Fig. S9d). The carbamylation (Supplementary Fig. S9e) and activity (Supplementary Fig. S9f) of mice GDH were decreased by BHB in WT but not in *Sirt4*^*–/–*^ mouse brain. Moreover, H3K27Ac (Supplementary Fig. S9g) and SIRT4 and GAD1 protein levels (Supplementary Fig. S9e) were increased by BHB. These are all consistent with the findings that BHB elevates H3K27Ac to overexpress SIRT4 and GAD1 and shift GABA/glutamate ratio in the brains.

Inactivation of GDH through SIRT4-catalyzed decarbamylation may account for the inability to relieve PTZ-induced epilepsy in *Sirt4*^*–/–*^ mice. We used epigallocatechin-3-gallate (EGCG), an inhibitor of GDH^47^, to reverse *Sirt4* knockout effects, i.e., preserving and directing glutamate to GABA. Oral gavage of EGCG (100 mg/kg) and BHB (500 mg/kg) for 4 weeks^48^, which had no impact on body weight (Supplementary Fig. S10a) and food intake (Supplementary Fig. S10b) of *Sirt4*^*–/–*^ mice, helped BHB to increase GABA and glutamate levels (Fig. 7f) and the GABA/glutamate ratio (Fig. 7g). It also led to remission of epileptic symptoms, which included a slower onset of the first seizure after PTZ administration (Fig. 7h), reduced intensity (Fig. 7i) and frequency of seizures (Fig. 7j), and shorter duration of seizures (Fig. 7k). These were consistent with the findings of the video/EEG for PTZ-induced *Sirt4*^*–/–*^ mice (Supplementary Fig. S10c). Lastly, the seizure-relieving efficacy of BHB was not observed in PTZ-treated *Sirt4-cKO* mice (Supplementary Fig. S10d–j). These findings collectively support the notion that high GABA/glutamate ratio and GABA levels in central nerve systems induced by BHB are required to relieve epilepsy.

## Discussion

Our results revealed that the KD-produced BHB accounts for antiepileptic efficacy of the KD. By inhibiting HDAC1/HDAC2, BHB increases H3K27Ac levels and facilitates the transcription of *SIRT4* and *GAD1*, which function synergistically to inhibit neuronal activation. By upregulating SIRT4, the decarbamylase of GDH, BHB inactivates GDH and accumulates glutamate for GABA production. Moreover, upregulation of GAD1 by BHB favors the production of the inhibitory neurotransmitter GABA from glutamate and increases the GABA/glutamate ratio to inhibit epilepsy (Fig. 7l)^49^. Notably, the activation of both SIRT4 and GAD1 seems to be essential for BHB to exert antiepileptic effects, as BHB exhibits limited antiepileptic potency in *Sirt4*^*–/–*^ mice although BHB increased brain GABA in both WT and *Sirt4*^*–/–*^ mice brains. Inhibiting GDH alone to proportionally increase glutamate and GABA also has limited antiepileptic potency. However, the GDH inhibitor EGCG potentiates BHB to inhibit epilepsy in *Sirt4*^*–/–*^ mice. This design suggests that a sufficiently high level of GABA is required in addition to high GABA/glutamate ratio to accomplish neuronal inhibition, given the array of other excitatory neurotransmitters besides glutamate that activate neurons^50,51^. It is worth to note that this mechanism may be related to mammal physiology. BHB and/or ketone bodies may be fat-sufficient signals, which inhibit neuronal excitation, required for hunting in mammals^52^, to keep energy expenditure in check and therefore exert antiepileptic efficacy. Therefore, it makes physiologic sense that KD and BHB inhibit epilepsy. KD induces cardiac fibrosis^53^ as high fat diet do^54^, supports that KD may generate fat-sufficient signals.

BHB is an endogenous and specific inhibitor of class I HDACs^40^. Moreover, GAD1 is down-regulated by HDAC1/HDAC2-mediated histone de-acetylation^42^. These results supported the upregulation of *GAD1* by BHB or KD. The identification of BHB that upregulates *SIRT4* in the mouse brains adds important clues to how BHB, whose production is upregulated by the KD, exerts antiepileptic effects. Therefore, in addition to its ADP-ribosyltransferase^30^, lipoamidase^55^ and deacylase^56^ activities, the newly identified de-carbamylase activity of SIRT4^31^ expands its roles in glutamate and GABA metabolism and neuronal activity regulation.

In addition to many mechanisms that had been proposed to explain the use of KD to constrain epilepsy, the identification of BHB as a major antiepileptic metabolite in KD is clinically noteworthy. As KD has been used as a dietary treatment for refractory epilepsy since the 1920s, side effects include poor taste and low compliance^57,58^, and prolonged KD administration tends to cause gastrointestinal disturbances and vomiting^59^, malnutrition^60^, cardiovascular diseases^61^, kidney stones^62^, and poor growth^63^. However, these limitations may be overcome by employing BHB or its efficacy-optimized molecules to replace the KD, which have fewer limitations in terms of toxicity and high doses.

Overall, our study highlights that BHB may serve as fat-rich signals to inhibit neuronal excitation. Moreover, the identification of BHB as an antiepileptic molecule has translational potential for the development of low toxic and high tolerable antiepileptic drugs. We were unable to obtain samples from human patients, mechanistic studies were performed on cultured cells, and in vivo studies were performed on mice model. Given that we study the GABA and glutamate homeostasis on epilepsy, we used non-competitive GABA(A) receptor inhibitor PTZ to generate mouse epilepsy model, which simulates the generalized symptoms of clinical human myoclonic epilepsy and is used to screen anti-epilepsy drugs. However, PTZ model fails to find the anti-non-spasmodic seizure effect of lamotrigine and tiagabine, and other epilepsy models, especially genetic animal epilepsy models are necessary to be employed to confirm whether KD and/or BHB will exert general anti-epilepsy efficacies.

## Materials and methods

### Cell culture

HEK293T (human embryonic kidney), HeLa (human adenocarcinoma), U87MG (human glioblastoma), and Neuro-2a (mouse neuroblastoma) cells were cultured in normal DMEM (Gibco) supplemented with 10% fetal bovine serum (FBS) (Gibco), 100 U/mL penicillin (Invitrogen) and 100 μg/mL streptomycin (Invitrogen). All cells were cultured an incubator at 37 °C and 5% CO_2_. Cell treatments are carried out by adding chemicals to the culture media to the levels specified in legends. BHB treatment takes 12 h.

### Animal experiments

Four-week-old C57BL/6 *Sirt4*^*–/–*^ mice (constructed using CRISPR-mediated deletion of exons 3 and 4 of the mouse *Sirt4* gene) were purchased from GemPharmatech Co., Ltd. (Shanghai, China). Genotypes were determined via PCR using genomic DNA obtained from tails and two primer pairs (sequences are provided in Supplementary Table S1). All mice were housed in a specific pathogen-free facility at 20–22 °C on a 12 h light/dark cycle with ad libitum access to food and water. All experiments were started when mice were 6 weeks old.

*Sirt4-cKO* mouse was generated based on C57BL/6 *Sirt4*^*flox/flox*^ mice. 2–3-month-old C57BL/6 *Sirt4*^*flox/flox*^ mice, purchased from Shanghai Research Center of the Southern model organisms, were anesthetized with a mixture of ketamine (100 mg/kg) and xylazine (10 mg/kg). Thirty three-gauge syringe needles (Hamilton, Reno, Nevada) were used to bilaterally infuse 1.0 μL of AAV virus into mouse dorsal hippocampus (AP = *–*2.0, ML = ±1.5, and DV = *–*2.0 from Bregma (mm)) or 0.5 μL of AAV virus into nucleus accumbens (NAc) (AP = +1.5, ML = + 1.6, and DV = *–*4.4 from Bregma (mm) at 10°) at a rate of 0.2 μL (0.1 μL for NAc) per min and the needle remained in place for 5 min before removal to prevent backflow. Intra-hippocampal microinjection of AAV vectors containing the gene for *Sirt4* or GFP alone were prepared by Genechem, Shanghai, China. AAV-injected mice were allowed to recover for 21 days before behavioral testing.

Carbon dioxide asphyxiation was adapted to euthanize mice. The animals were brought from their home cages to the CO_2_ euthanasia apparatus in the procedure room. Remove the filter top from the cage, and cover the cage with the stainless-steel euthanasia lid. Turn on the flow of CO_2_ to the cage by using the flow meter attached to the pressure regulator. Expose the animals to CO_2_ until complete cessation of breathing for a minimum of 2 min after 5 to 10 min CO_2_ exposing. Continue the following experiments after visualizing all mice in the cage were euthanized.

Adult male mice were euthanized, and serum and brain tissues were collected according to standard procedure. Briefly, brains were perfused with saline to remove blood cells before isolation, and the tissues were quickly removed and snap frozen in liquid nitrogen for further analyses. All animal procedures were conducted in accordance with the animal care committee at Fudan University, Shanghai, China.

### Mouse KD or BHB treatment

Eight-week-old WT and *Sirt4*^*–/–*^ mice received an ND (Bio-Serv, F0761) or KD (Bio-Serv, F3666) with free access to drinking water for 12 weeks. BHB ester (HVMN, 200 µL by oral gavage daily, 80 mg per mouse) treatments to mice last 4 weeks before analysis.

### PTZ-induced acute seizure mouse model

Twenty-week-old male mice received a single dose of intraperitoneal injection of PTZ (Sigma), with the dosage ranging between 40 and 45 mg/kg of body weight after being fed ND or KD. Mice were observed for 10 min after each injection.

The scores of seizure were staged as follows: 0 grade: no response; I grade: facial movements, including blinking, twitching, rhythmic chewing, etc; II grade: I grade plus rhythmic nodding; III grade: II grade plus forelimb clonic; IV grade: III grade plus standing on hind legs; V grade: IV grade plus falling.

### Mouse EEG

Under Ketamine/Xylazine anesthesia, chronic EEG electrodes were surgically implanted using aseptic technique in mice. Micro-screws anchored into the cranium served as epidural electrodes and were connected to a microplug adhered to the cranium with dental acrylic. Recording electrodes were positioned bilaterally over each hemisphere (anterior-posterior, –2.0 mm from bregma; lateral, ±1.75 mm); a reference electrode was placed anterior to bregma (anterior-posterior, +0.5 mm) and a ground electrode was placed over the cerebellum. Mice were allowed to recover for 5–7 days before recording. The Telefactor Video/EEG Monitoring System was used to record EEGs and to videotape behavior simultaneously from un-anesthetized free-moving mice. The low-frequency filter setting was 0.3 Hz, and the high-frequency filter was set at 70 Hz.

### CRISPR/Cas9-mediated deletion of *HDAC1* and *HDAC2*

*HDAC1*-knockout and *HDAC2*-knockout HeLa were generated by a CRISPR/Cas9 plasmid, respectively targeting *HDAC1* and *HDAC2*. The plasmids were constructed by cloning the annealed sgRNA into px459 vector. The sgRNAs were designed by CRISPR Design website (http://crispr.mit.edu) and the sequences are listed in Supplementary Table S1.

### Plasmid construction

The cDNA encoding full-length human *SIRT3*, *SIRT4*, *SIRT5*, *GDH*, *GAD1*, *BDH1*, *CPS1*, *HDAC1,* and *HDAC2* was cloned into Flag, Myc, HA-tagged vectors (pcDNA3.1-Flag; pcDNA3.1-Myc; pcDNA3.1-HA). Point mutations of *GDH* were generated by Mut Express II Fast Mutagenesis Kit V2 (Vazyme).

### Metabolomics analysis

#### Metabolite extraction

Frozen tissues were homogenized in 80% methanol (methanol:water = 4:1) and centrifuged at 14,000× *g* for 15 min (4 °C). The supernatant was transferred to a new tube and lyophilized into a pellet. The sample was resuspended in 1500 μL of extract solution (acetonitrile:methanol:water = 2:2:1, with isotopically labelled internal standard mixture). After 30 s of vortexing, samples were sonicated for 10 min in an ice-water bath. Next, the samples were incubated for 1 h at –40 °C and centrifuged at 12,000× *g* for 15 min at 4 °C. The resulting supernatant was transferred to a fresh glass vial for analysis.

#### LC-MS/MS analysis

LC-MS/MS analyses were performed using a UHPLC system (Vanquish, ThermoFisher Scientific) with an UPLC BEH Amide column (2.1 mm × 100 mm, 1.7 μM) coupled to a QExactive HFX mass spectrometer (Orbitrap MS, ThermoFisher Scientific). The mobile phase consisted of 25 mmol/L ammonium acetate and 25 mmol/L ammonia hydroxide in water (pH = 9.75) (A) and acetonitrile (B). The analysis was carried out with an elution gradient as follows: 0–0.5 min, 95% B; 0.5–7.0 min, 95%–65% B; 7.0–8.0 min, 65%–40% B; 8.0–9.0 min, 40% B; 9.0–9.1 min, 40%–95% B; 9.1–12.0 min, 95% B. The column temperature was 30 °C. The temperature of the autosampler was 4 °C, and the injection volume was 2 μL.

The QE HFX mass spectrometer was used due to its ability to acquire MS/MS spectra in information-dependent acquisition mode under the control of acquisition software (Xcalibur, ThermoFisher Scientific). In this mode, the acquisition software continuously evaluated the full-scan MS spectrum. The ESI source conditions were set as follows: sheath gas flow rate of 30 Arb, Aux gas flow rate of 25 Arb, capillary temperature of 350 °C, full MS resolution of 60,000, MS/MS resolution of 7500, collision energy of 10/30/60 in NCE mode, and spray voltage of 3.6 kV (positive) or –3.2 kV (negative).

#### Data analysis

Raw data were converted to the mzXML format using ProteoWizard and processed using an in-house programme, which was developed using R and based on XCMS, for peak detection, extraction, alignment, and integration. An in-house MS2 database (BiotreeDB) was applied for metabolite annotation. The cut-off for annotation was set at 0.3.

#### ^13^C labelling of metabolites

Cells were plated in 10-cm dishes for 24 h before labelling. The next day, the medium was changed for unlabelled medium without glutamine 1 h before adding the ^13^C_5_-glutamine medium. Cells were incubated in labelling media containing 2 mM ^13^C_5_-glutamine (Sigma) for 6 h before collection. At the time of collection, cells were washed with prechilled saline, lyzed with (40%) methanol:(40%) acetonitrile:(20%) water with 0.1 M formic acid, and processed as described above (Metabolomics). Metabolites were reconstituted in 100 μL of 0.1% formic acid in LC/MS-grade water, vortex-mixed, and centrifuged to remove debris. The supernatants were analyzed using LC-MS/MS.

### Proteomics analysis

#### Protein digestion

The procedures for protein digestion were performed according to the filter-aided sample preparation (FASP) technique^64^ with slight modifications. Briefly, FASP digestion was adapted for the following procedures in Microcon PL-10 filters. Following a three-time buffer displacement with 4 M guanidine hydrochloride and 100 mM Tris-HCl, pH 8.0, proteins were reduced using 10 mM DTT at 37 °C for 30 min, followed by alkylation with 30 mM iodoacetamide at 25 °C for 45 min in the dark. Digestion was carried out with trypsin (enzyme:protein = 1:50) at 37 °C overnight after washing with 20% acetone nitrile and digestion buffer (30 mM Tris-HCl, pH 8.0). After digestion, the solution was filtrated out, the filter was washed with 15% ACN, and all filtrates were pooled, vacuum-dried, and kept at –80 °C until use.

#### Carbamyl peptide enrichment

Carbamyl peptide enrichment was performed using pan-anti-carbamyl lysine antibody (Abcam, Cat# ab175132) conjugated with protein A beads (Sigma, Cat# 16-156). Peptides were mixed with antibody-conjugated beads at 4 °C for 6–8 h under rotary shaking. The mixture was briefly centrifuged at 1000× *g* (< 10 s), and the supernatant was carefully removed. Antibody-conjugated beads were subsequently washed thrice with phosphate buffered saline (PBS) buffer. Finally, the bound carbamyl peptides were eluted with 30% ACN/0.1% TFA and concentrated in a SpeedVac. Before MS analysis, all carbamyl peptides were desalted.

#### LC-MS/MS analysis

LC-MS analysis was performed using a nanoflow EASYnLC 1200 system (ThermoFisher Scientific, Odense, Denmark) coupled to an Orbitrap Fusion Lumos mass spectrometer (ThermoFisher Scientific, Bremen, Germany). A one-column system was adopted for all analyses. Samples were analyzed on a C18 analytical column (75 µm i.d. ×25 cm, ReproSil-Pur 120 C18-AQ, 1.9 µm (Dr. Maisch GmbH, Germany)) constructed in-house. The mobile phases consisted of Solution A (0.1% formic acid) and Solution B (0.1% formic acid in 80% ACN). The derivative peptides were eluted using the following gradients: 2%–5% B in 2 min, 5%–35% B in 100 min, 35%–44% B in 6 min, 44%–100% B in 3 min, 100% B for 10 min, at a flow rate of 200 nL/min. Data-dependent analysis was employed in MS analysis: the time between master scan was 3 s, and fragmented in HCD mode, normalized collision energy was 30.

#### Data processing and analysis

Raw MS files were searched using Protein Discoverer (version 2.4, ThermoFisher Scientific) with Mascot (version 2.7.0, Matrix Science). Data were processed using the UniProt mouse protein database (55,462 entries). The mass tolerances were 10 ppm for precursor and fragment mass tolerance 0.05 Da. Up to two missed cleavages were allowed. The search engine set protein N-acetylation, pyroglutamate on peptide N termini, oxidation on methionine, and carbamylation on Lys as variable modifications and cysteine carbamidomethylation as a fixed modification.

### Recombinant SIRT4 expression and purification

Recombinant SIRT4 expression was performed as previously described with slight modifications^65^. The NAD^+^-dependent deacetylase domain encompassing residues 27–314 of SIRT4 was subcloned into a pSUMOH10 vector (modified based on pET28b) containing an N-terminal 10× HIS-SUMO tag. SIRT4^27–314^ were expressed in *Escherichia coli* BL21 (DE3) strain (Transgene, China). After induction with 0.2 mM isopropyl β-D-thiogalactoside at 16 °C in LB medium supplemented with 0.1 mM ZnCl_2_ overnight, cells were harvested by centrifugation at 4000× *g* for 20 min at 4 °C. The pellets were resuspended in buffer A containing 20 mM Tris-HCl, pH 7.5, 500 mM NaCl, 5% glycerol, and 20 mM imidazole and were then disrupted using an EmulsiFlex-C3 homogenizer (Avestin) at 4 °C. The lysate was further cleared by centrifugation at 14,000× *g* for 60 min at 4 °C, and the supernatant was loaded onto a His-trap affinity column (GE Healthcare). After washing with buffer A, bound proteins were subjected to on-column cleavage by ULP1 SUMO protease. The tag-free SIRT4^27–314^ protein was collected as flow-through, centrifuge-concentrated, and then purified on an anion-exchange QHP column followed by size exclusion chromatography on a Superdex 75 column (GE Healthcare). Purified peak fractions were pooled, concentrated, aliquoted, and stored at −80 °C for future use.

### MS-based in vitro decarbamylation assays

The ability of SIRT4 to hydrolyze carbamyl lysine was measured using MALDI TOF/TOF. Carbamyl peptide (200 µM) was incubated with 5 µM SIRT3, SIRT4, or SIRT5 in reaction buffer containing 50 mM Tris-HCl, pH 7.8, 1 mM DTT, and 1 mM MgCl_2_ without or with 2 mM NAD^+^ for 2 h at 37 °C. Before MS analysis, all carbamyl peptides were desalted and eluted using C18 tips. Samples were analyzed using a 4800 Plus MALDI TOF/TOF Analyzer (Applied Biosystems/MDSSCIEX) and operated in Reflector Positive mode using 4000 Series Explorer Software. The laser intensity was set at 3500 V.

### SDS-PAGE and western blotting assay

SDS-PAGE and western blotting assay were performed following standard protocols. Briefly, cells were harvested with loading buffer containing 50 mM Tris-HCl pH 6.8, 10% glycerol (v/v), 2% SDS (w/v), 4% β-mercaptoethanol (v/v), and 0.0012% bromophenol blue (w/v). For western blotting analysis, each sample was subjected to SDS-PAGE and transferred to nitrocellulose membranes (GE Healthcare Life Science). The membranes were blocked in 5% (w/v) skim milk in Tris-buffered saline with 0.1% (v/v) Tween-20 (TBST) for 1 h at room temperature and were then probed with primary antibodies in antibody dilution buffer (QuickBlock^TM^, Beyotime) at 4 °C overnight. After incubation with horseradish peroxidase-conjugated secondary antibodies in TBST (containing 5% skim milk), membranes were developed using ECL-Plus (Thermo Fisher Scientific) and visualized using Typhoon (GE Healthcare Life Science).

### Protein half-life assays

Cells were treated under the indicated conditions. For half-life studies, cycloheximide (100 mg/mL, Sigma) was added to the medium. Cells were collected at the indicated time points and protein concentrations were measured, then analyzed by immunoblot analysis.

### ChIP assays

ChIP assays were conducted using the ChIP Assay Kit (Beyotime). First, U87MG and HeLa cells treated with or without BHB were crosslinked by 1% formaldehyde for 10 min. DNA was then sonicated into fragments with a mean length of 200–500 bp, and sheared chromatin was immunoprecipitated with antibodies against H3K27Ac (Abcam) or non-specific rabbit IgG (Santa Cruz) overnight at 4 °C. The precipitated DNA fragments were then identified by PCR and quantified by quantitative real-time PCR (qRT-PCR).

### RNA isolation and qRT-PCR analyses

RNA was extracted from cells using TRIzol (Transgene, China) according to the manufacturer’s instructions, and the concentration/purity were determined using NanoDrop Spectrophotometer (ThermoFisher Scientific). cDNA was synthesized using the HiScript III 1st Strand cDNA Synthesis Kit (Vazyme) according to the manufacturer’s instructions. qRT-PCR was performed with SYBR qPCR Master Mix (Vazyme) on a Real-Time Thermocycler (BioRad). qPCR analyses in cells or tissues are relative to β-actin. The primer sequences are listed in Supplementary Table S1.

### Mitochondrial isolation

Cells were washed with prechilled PBS, harvested with isolation buffer (20 mM HEPES, pH 7.4, 140 mM KCl, 10 mM EDTA, 5 mM MgCl_2_ with a protease and phosphatase inhibitor cocktail), and homogenized using a chilled glass-Teflon homogenizer with 20 strokes. The homogenate was centrifuged twice at 700× *g* for 10 min at 4 °C. The supernatant was transferred to a new tube and centrifuged at 9000× *g* for 30 min at 4 °C to obtain the mitochondrial pellet. The pellet was collected and washed thrice with washing buffer (20 mM HEPES, pH 7.4, 800 mM KCl, 10 mM EDTA, and 5 mM MgCl_2_).

### Histone extraction

Cells were washed twice with PBS and resuspended in 200 μL of buffer A (10 mM HEPES (pH 7.9), 10 mM KCl, 1.5 mM MgCl_2_, 0.34 M sucrose, 10% glycerol, 1 mM DTT, 0.1% Triton X-100, and protease inhibitor mixture (Roche Molecular Biochemicals). After incubation for 5 min on ice, the nuclei were collected in the pellet by low-speed centrifugation (1500× *g*, 4 min, 4 °C). The nuclei were washed once with buffer A without 0.1% Triton X-100 and then lysed in 200 μL of buffer B (3 mM EDTA, 0.2 mM EGTA, 1 mM DTT, and protease inhibitor mixture). After 10 min incubation on ice, soluble histones were separated from chromatin by centrifugation (2000× *g*, 4 min).

### RNA interference

Small RNA interference and stable knockdown were used in this study. In RNA interference, double-stranded siRNAs targeting *GDH* and *BDH1*, respectively, were purchased from GenePharma and transfected into cells via RNAiMax (Invitrogen) according to the manufacturer’s instructions. siRNA transfections were performed with Lipo2000 (Life technology) according to the manufacturer’s instruction. Cells were cultured in six-well plate (ThermoFisher Scientific) and siRNAs were used at a concentration of 20 nM. Cells were harvested at 48 h post-transfection.

Stable shRNA knockdown cells were generated by co-transfecting cells with pCMV-VSV-G, pCMV-Gag-Pol, and shRNA plasmids by the calcium phosphate method. DMEM containing 10% FBS was used to culture the cells for 6 h after the transfection. After 24 h of transfection, supernatant of the cultured medium was collected and used as retrovirus preparation to infect cells at a density of 10% confluence in 90 mm dishes. Cells were re-infected 48 h after the initial infection using 5 μg/mL puromycin (Amresco) for selecting cells. All the sequences of siRNA/shRNA are listed in Supplementary Table S1. The knock-down efficiency was verified by qPCR or western blotting assay.

### Measurement of GDH enzyme activity

Fresh tissues were weighed and transferred to solubilization buffer (1% Triton X-100, 150 mM NaCl, 10 mM KCl, 5 mM EDTA, 50 mM Tris-HCl, pH 8.0) containing protease and phosphatase inhibitors in 5 µL of extraction buffer per mg tissue and homogenized using ceramic beads. The samples were centrifuged at 14,000× *g* for 15 min (4 °C). The supernatant was transferred to a new tube for immunoprecipitation and enzymatic activity measurements, and protein concentrations were determined using a BCA protein assay kit (Beyotime).

The enzyme activity of GDH were determined by using a Glutamate Dehydrogenase Activity Assay Kit (Sigma). In short, the GDH protein was mixed with the assay buffer and incubated for 2 min at 37 °C in darkness. The change in absorbance at 450 nm was measured every 30 s for 10 min at 37 °C in a SpectraMax M5 Microplate Reader (Molecular Devices). Each sample was analyzed in triplicate, and the enzyme activity of GDH was calculated following the manufacturer’s instruction.

### In vitro GAD1 enzymatic assay

Pre-warmed (37 °C) assay buffer (50 mM KPO_4_ pH 8.0, 0.2 mM PLP, 1 mM AET, 100 μg/mL BSA) containing 20 mM sodium glutamate was added to test tube containing the different cell lysates in ratio 1:1. After 120 min of incubation at 37 °C, samples were immediately placed in ice to stop the reaction and GABA was immediately quantified. Each determination was performed in triplicates and the whole experiments were repeated twice.

### BHB treatment and measurement

Mice were administered with BHB esters (HVMN), 200 µL by oral gavage daily, 80 mg per mouse.

Levels of BHB in serum and brain were determined by using a BHB Assay Kit (Sigma) according to the manufacturer’s instruction. In short, the samples were mixed with the assay buffer and incubated the reaction for 30 min at room temperature in darkness. Absorbance was measured at 450 nm (A450) in a SpectraMax M5 Microplate Reader (Molecular Devices). Each sample was analyzed in triplicate.

### Measurements of the concentrations of AcAc and acetone

Serum and brain AcAc levels were measured using the Ketone Body Assay Kit (Abnova, KA1630) according to the manufacturer’s instructions. The acetone concentrations were measured using the Goat Acetone Elisa Kit (MyBioSource, MBS9310393) according to the manufacturer’s instructions.

### Immunofluorescence analysis

Animals were deeply anesthetized with vaporized sevoflurane and perfused with 50 mL saline, followed by 50 mL 4% PFA. The brains were extracted and soaked in 4% PFA at 4 °C overnight, cryoprotected by transferring to 30% sucrose solution until the brains were saturated. Coronal brain sections (40 µm) were cut using a freezing microtome (CM1950, Leica). The slices undergoing immunohistochemical staining were washed in PBS three times (5 min each time) and pre-incubated in PBS containing 5% normal goat serum and 0.3% Triton X-100 for 1 h at room temperature, before incubating with the following primary antibodies at 4 °C overnight: rabbit anti-Cre recombinase (1:1000, 15036, Cell Signaling, USA). Then, the sections were washed in PBS three times (5 min each time) and incubated with a secondary antibody at 37 °C for 1 h. The slices were washed in PBS thrice (5 min each time). Finally, all of the slices were mounted onto glass slides, dried, and covered with Antifade Mounting Medium (Solarbio, S2100, China).

### Quantification and statistical analysis

Statistical tests were performed using GraphPad Prism 8 software and two-tailed unpaired Student’s *t*-test was used to compare two groups. All data are presented as the means ± standard deviation (SD). *P* values were calculated assuming a normal distribution and were corrected for multiple hypotheses using the Benjamini–Hochberg procedure. *P* values are denoted in figures as: ns, *P* > 0.05; *P < 0.05; **P < 0.01; ***P < 0.001; ****P < 0.0001.

### Supplementary information

Supplementary information
