# Author Correction: Ketogenic diet-produced β-hydroxybutyric acid accumulates brain GABA and increases GABA/glutamate ratio to inhibit epilepsy

**DOI:** 10.1038/s41421-024-00669-w

**Published:** 2024-04-30

**Authors:** Ya-Nan Qiao, Lei Li, Song-Hua Hu, Yuan-Xin Yang, Zhen-Zhen Ma, Lin Huang, Yan-Peng An, Yi-Yuan Yuan, Yan Lin, Wei Xu, Yao Li, Peng-Cheng Lin, Jing Cao, Jian-Yuan Zhao, Shi-Min Zhao

**Affiliations:** 1 The Obstetrics & Gynaecology Hospital of Fudan University, State Key Laboratory of Genetic Engineering, Shanghai Key Laboratory of Metabolic Remodelling and Health, Institutes of Biomedical Sciences, and Children’s Hospital of Fudan University, Fudan University, Shanghai, China; 2 Department of Anatomy, School of Basic Medical Sciences, Zhengzhou University, Zhengzhou, Henan China; 3 Department of Cell Biology, Blavatnik Institute, Harvard Medical School, Boston, MA USA; 4 Key Laboratory for Tibet Plateau Phytochemistry of Qinghai Province, College of Pharmacy, Qinghai University for Nationalities, Xining, Qinghai China; 5 Institute for Developmental and Regenerative Cardiovascular Medicine, MOE-Shanghai Key Laboratory of Children’s Environmental Health, Xinhua Hospital, Shanghai Jiao Tong University School of Medicine, Shanghai, China

**Keywords:** Post-translational modifications, Cancer metabolism, Mechanisms of disease

Correction to: *Cell Discovery* (2024) 10:17

10.1038/s41421-023-00636-x published online 13 February 2024

In this article, the authors have realized that two corrections need to be made in the affiliations.

1. Lei Li should be credited as co-first author of the paper. A sentence stating that “These authors contributed equally: Ya-Nan Qiao, Lei Li.” has been added.
2. The corresponding author Shi-Min Zhao is not associated with affiliation 3, Department of Cell Biology, Blavatnik Institute, Harvard Medical School, Boston, MA, USA.

