# Supplementary material for: Ketogenic diet-produced β-hydroxybutyric acid accumulates brain GABA and increases GABA/glutamate ratio to inhibit epilepsy
Source: Cell Discov. 2024 Feb 13;10:17. doi: 10.1038/s41421-023-00636-x (PMC10861483; doi:10.1038/s41421-023-00636-x)
Supplement: Supplementary file 1 — Supplementary information [file 41421_2023_636_MOESM1_ESM.pdf]

Qiao et al.,

Ketogenic Diet produced β-Hydroxybutyric Acid Accumulates Brain GABA and Increases GABA/Glutamate Ratio to Inhibit Epilepsy

Supplemental Figures

Fig.S1

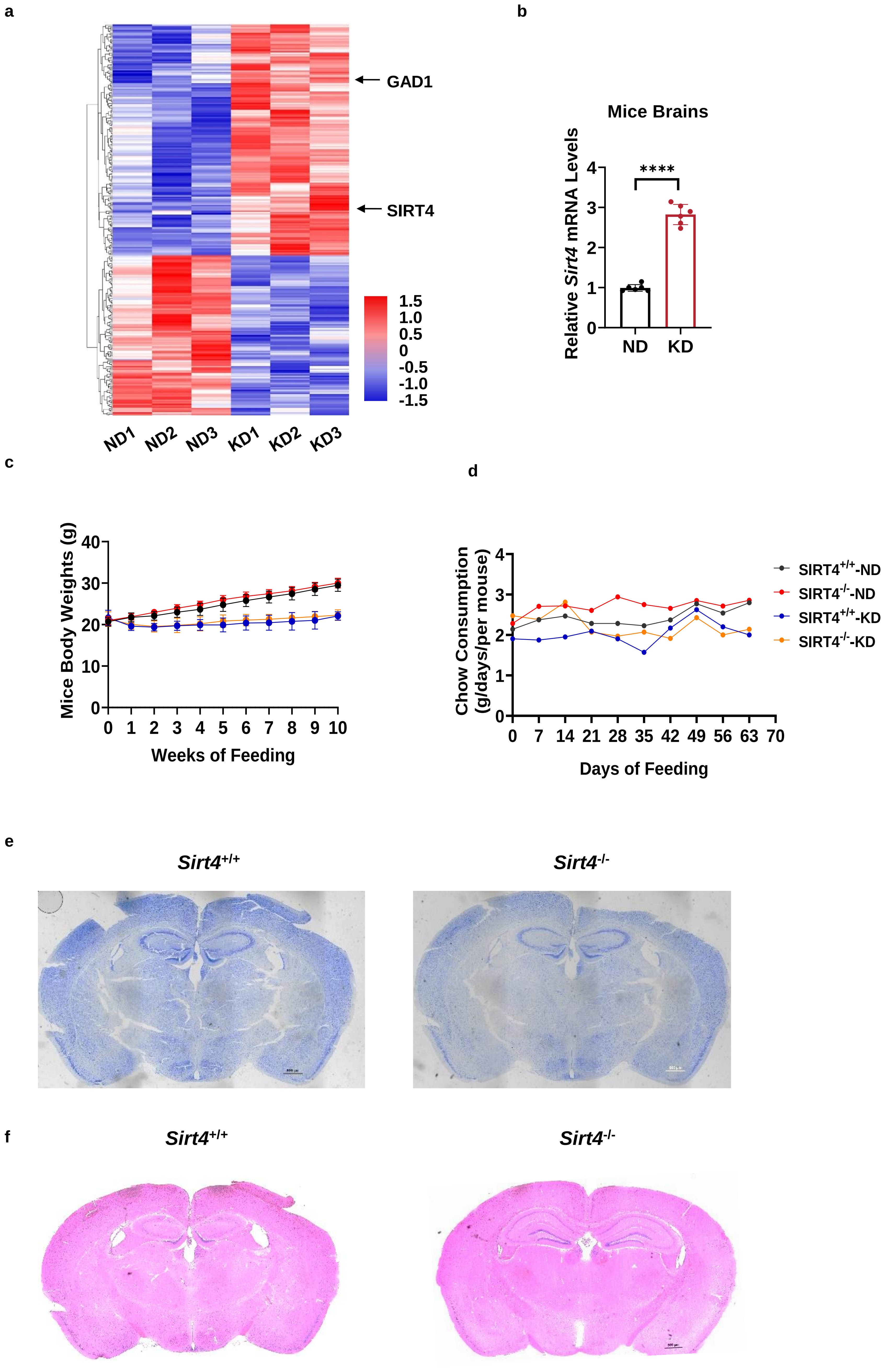
a
GAD1
SIRT4
1.5
1.0
0.5
0
-0.5
-1.0
-1.5
ND1
ND2
ND3
KD1
KD2
KD3
b
Mice Brains
Relative Sirt4 mRNA Levels
****
0
1
2
3
4
ND
KD
c
Mice Body Weights (g)
0
10
20
30
40
0 1 2 3 4 5 6 7 8 9 10
Weeks of Feeding
d
Chow Consumption (g/days/per mouse)
0
1
2
3
4
0 7 14 21 28 35 42 49 56 63 70
Days of Feeding
SIRT4+/+-ND
SIRT4-/--ND
SIRT4+/+-KD
SIRT4-/--KD
e
Sirt4+/+
Sirt4-/-
f
Sirt4+/+
Sirt4-/-

Fig. S1 *Sirt4$^{-/-}$* mice grew normally.

a KD induced gene expression alteration. Heatmap of differentially expressed genes in KD-fed mice brains compared to those in ND-fed mice brains. The increased expression of SIRT4 and GAD1 were marked. The histogram reflected the protein levels of SIRT4 and GAD1. n = 3 mice.

b KD increased SIRT4 transcription. SIRT4 levels in the brains of KD- and ND-fed mice were compared.

c-f Weights (**c**), food consumption (**d**), Nissl bodies in the brain (**e**) and the structure of the brain (**f**) were monitored for C57 and *Sirt4$^{-/-}$*mice that were fed either ND or KD.

Fig.S2

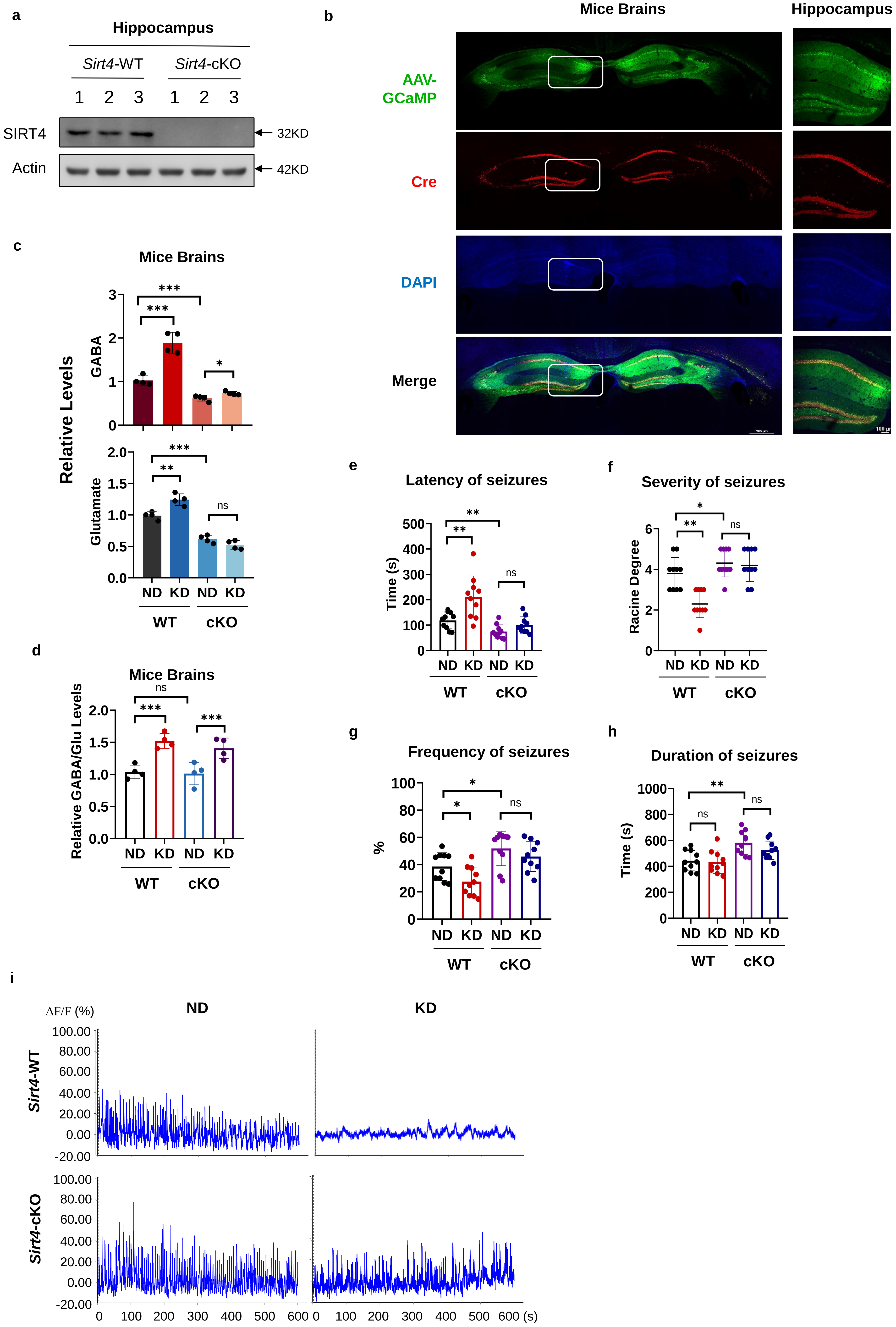
a
Hippocampus
Sirt4-WT
Sirt4-cKO
1 2 3 1 2 3
SIRT4
Actin
32KD
42KD
b
Mice Brains
Hippocampus
AAV-GCaMP
Cre
DAPI
Merge
c
Mice Brains
Relative Levels
GABA
Glutamate
ND KD ND KD
WT cKO
d
Mice Brains
Relative GABA/Glu Levels
ND KD ND KD
WT cKO
e
Latency of seizures
Time (s)
ND KD ND KD
WT cKO
f
Severity of seizures
Racine Degree
ND KD ND KD
WT cKO
g
Frequency of seizures
%
ND KD ND KD
WT cKO
h
Duration of seizures
Time (s)
ND KD ND KD
WT cKO
i
ΔF/F (%)
ND
KD
Sirt4-WT
Sirt4-cKO
0 100 200 300 400 500 600 (s)

Fig S2. Central nerve SIRT4 was required accounts for KD's anti-epileptic efficacies.

a-b The conditional knockout effect of *Sirt4* in the hippocampus by Cre-AAV was verified by WB (**a**) and immunofluorescence (**b**).

c-d KD increased GABA levels (**c**) and the GABA/glutamate ratio (**d**) in WT and *Sirt4*-cKO mouse brains. Data were represented as individual values.

e-i KD SIRT4-dependently relieved seizure phenotypes. The ND- and KD-fed WT and *Sirt4*-cKO mice were subjected to measurements after they were administered PTZ. Latency time (**e**), the highest level (**f**), the frequency (**g**), the duration of seizures (**h**) and video/EEG (**i**) were monitored. Data were mean ± s.d., n = 10 mice in each treatment.

Fig.S3

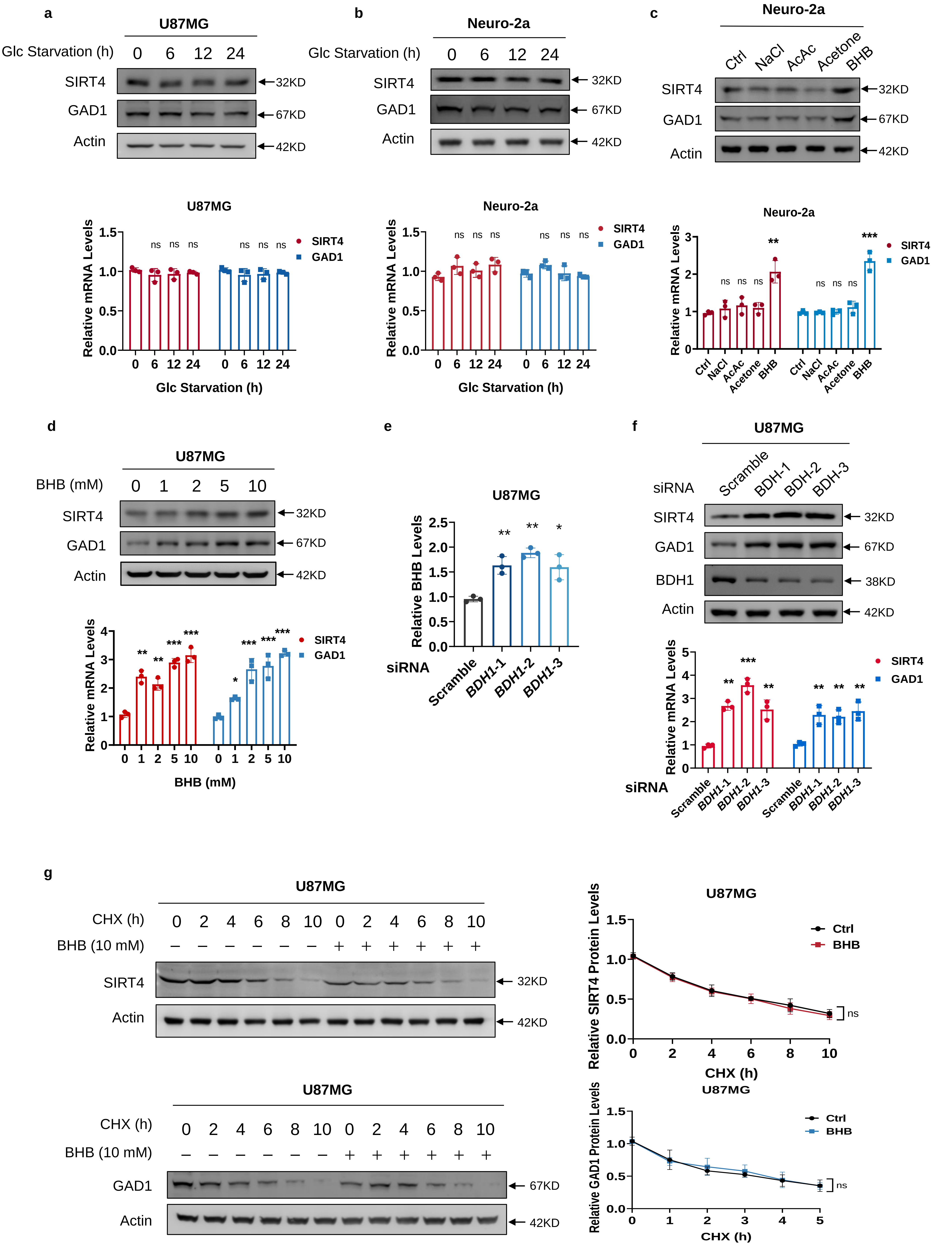
a
U87MG
Glc Starvation (h) 0 6 12 24
SIRT4 32KD
GAD1 67KD
Actin 42KD
b
Neuro-2a
Glc Starvation (h) 0 6 12 24
SIRT4 32KD
GAD1 67KD
Actin 42KD
c
Neuro-2a
Ctrl NaCl AcAc Acetone BHB
SIRT4 32KD
GAD1 67KD
Actin 42KD
Relative mRNA Levels
Glc Starvation (h)
SIRT4
GAD1
ns
d
U87MG
BHB (mM) 0 1 2 5 10
SIRT4 32KD
GAD1 67KD
Actin 42KD
BHB (mM)
e
U87MG
Relative BHB Levels
siRNA
Scramble BDH1-1 BDH1-2 BDH1-3
f
U87MG
siRNA Scramble BDH-1 BDH-2 BDH-3
SIRT4 32KD
GAD1 67KD
BDH1 38KD
Actin 42KD
g
U87MG
CHX (h) 0 2 4 6 8 10 0 2 4 6 8 10
BHB (10 mM) – – – – – – + + + + + +
SIRT4 32KD
Actin 42KD
GAD1 67KD
Relative SIRT4 Protein Levels
Relative GAD1 Protein Levels
CHX (h)
Ctrl
BHB
ns

Fig. S3 KD-induced BHB activated *SIRT4* and *GAD1* transcription.

a-b Low glucose had no impact on *SIRT4* and *GAD1* expression levels. The protein and mRNA levels of *SIRT4* and *GAD1* were determined at indicated times after U87MG (**a**) and Neuro-2a (**b**) cells were starved for glucose.

c BHB-induced *SIRT4* and *GAD1* expression. The protein and mRNA levels of *SIRT4* and *GAD1* were determined in Neuro-2a cells and in Neuro-2a cells cultured in NaCl-, AcAc-, acetone-, and BHB-containing media.

d BHB induced *SIRT4* and *GAD1* expression in a dose-dependent manner. Protein and mRNA levels of *SIRT4* and *GAD1* were determined in U87MG cells cultured in media supplemented with different levels of BHB.

e-f *BDH1* knockdown increased BHB levels and *SIRT4* and *GAD1*. *BDH1* was silenced with independent siRNAs in U87MG cells. The BHB levels were measured in U87MG cells (**e**). Protein and mRNA levels of *SIRT4* and *GAD1* were compared between siRNA-untreated and -treated cells (**f**).

g BHB did not affect *SIRT4* or *GAD1* degradation. *SIRT4* and *GAD1* protein levels were determined in U87MG cells with or without BHB treatment after their protein translation was inhibited by CHX for the indicated times (left). Quantification of triplicate results (right) was shown.

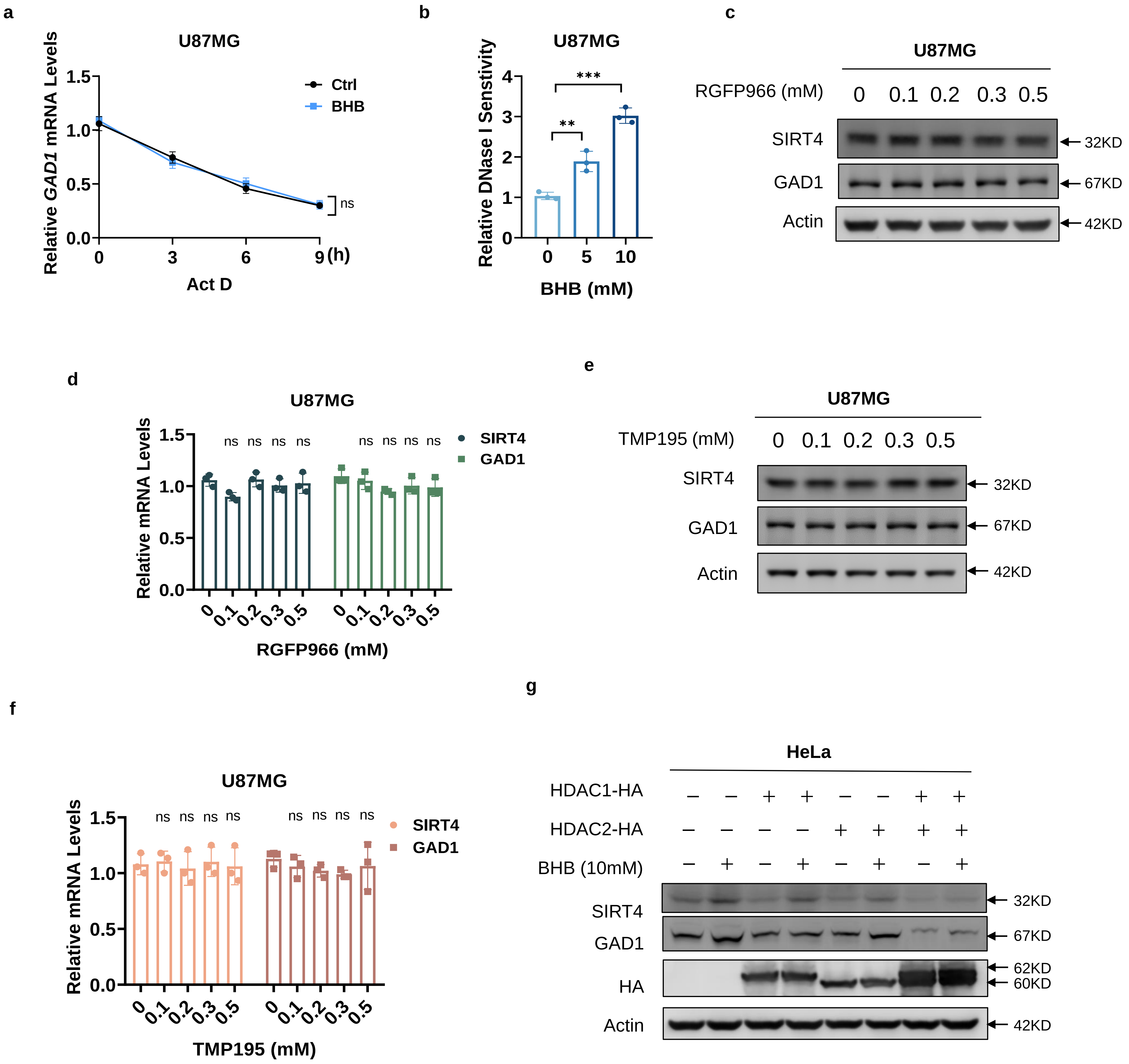
a
U87MG
Relative GAD1 mRNA Levels
Ctrl
BHB
ns
Act D
(h)
b
U87MG
Relative DNase I Senstivity
***
**
BHB (mM)
c
U87MG
RGFP966 (mM) 0 0.1 0.2 0.3 0.5
SIRT4 32KD
GAD1 67KD
Actin 42KD
d
U87MG
Relative mRNA Levels
ns ns ns ns ns ns ns ns
SIRT4
GAD1
RGFP966 (mM)
e
U87MG
TMP195 (mM) 0 0.1 0.2 0.3 0.5
SIRT4 32KD
GAD1 67KD
Actin 42KD
f
U87MG
Relative mRNA Levels
ns ns ns ns ns ns ns ns
SIRT4
GAD1
TMP195 (mM)
g
HeLa
HDAC1-HA
HDAC2-HA
BHB (10mM)
SIRT4 32KD
GAD1 67KD
HA 62KD 60KD
Actin 42KD

Fig. S4 BHB inhibited HDAC1/HDAC2 to activate *SIRT4* and *GAD1* transcription.

a mRNA degradation did not account for BHB-promoted *SIRT4* and *GAD1* mRNA increase. *GAD1* mRNA in U87MG cells was detected at various time points with 10 mM actinomycin D (Act D).

b BHB increased chromatin accessibility of the *GAD1* promoter. Quantitative PCR was performed on DNase I-pretreated nuclei of U87MG cells treated with BHB at the indicated concentrations.

c-f Effects of HDAC inhibitors on *SIRT4* and *GAD1* expression. Protein and mRNA levels of *SIRT4* and *GAD1* were measured in U87MG cells and in U87MG cells after treatment with RGFP966 (**c, d**) and TMP195 (**e, f**).

g *HDAC1/HDAC2* expression abrogated the ability of BHB to increase *SIRT4* and *GAD1* expression. *SIRT4* and *GAD1* expression in HeLa and HeLa cells overexpressing either *HDAC1*, *HDAC2*, or both were detected with and without BHB treatment.

Fig.S5

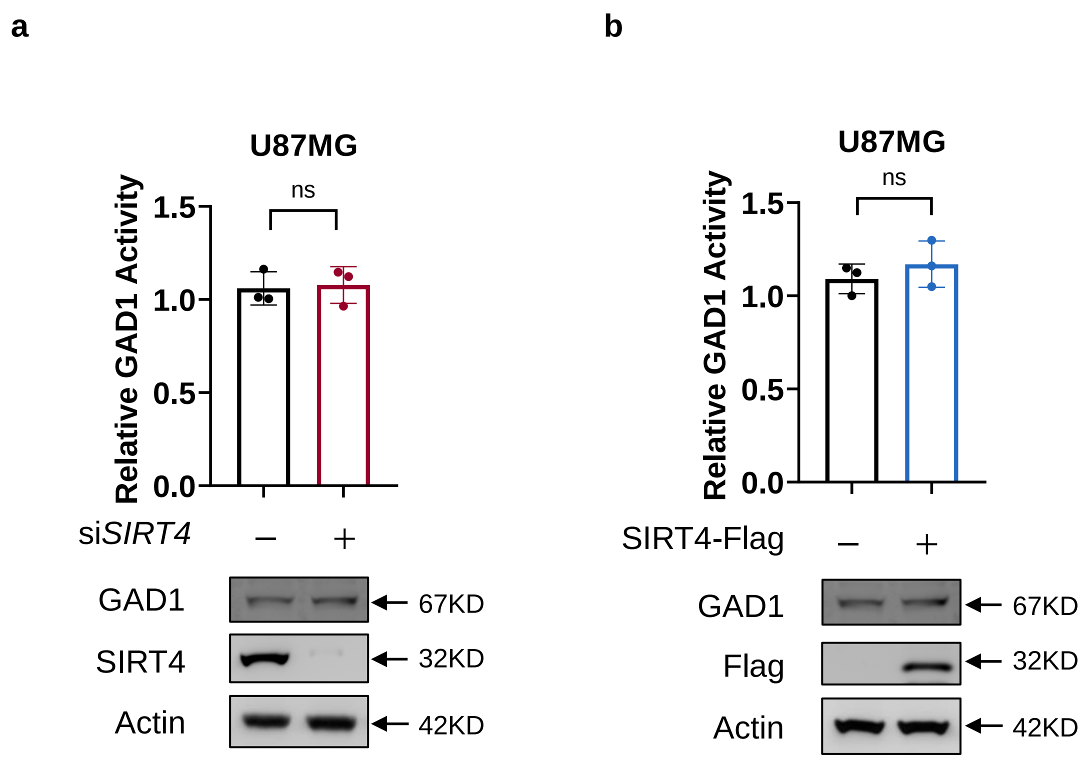
a
U87MG
ns
Relative GAD1 Activity
1.5
1.0
0.5
0.0
siSIRT4 – +
GAD1
SIRT4
Actin
67KD
32KD
42KD
b
U87MG
ns
Relative GAD1 Activity
1.5
1.0
0.5
0.0
SIRT4-Flag – +
GAD1
Flag
Actin
67KD
32KD
42KD

Fig. S5 GAD1 activity was unresponsive to SIRT4 variation.

a-b GAD1 activity was determined in both *SIRT4*-silenced (**a**) and *SIRT4*-overexpressing (**b**) U87MG cell lines.

Fig.S6

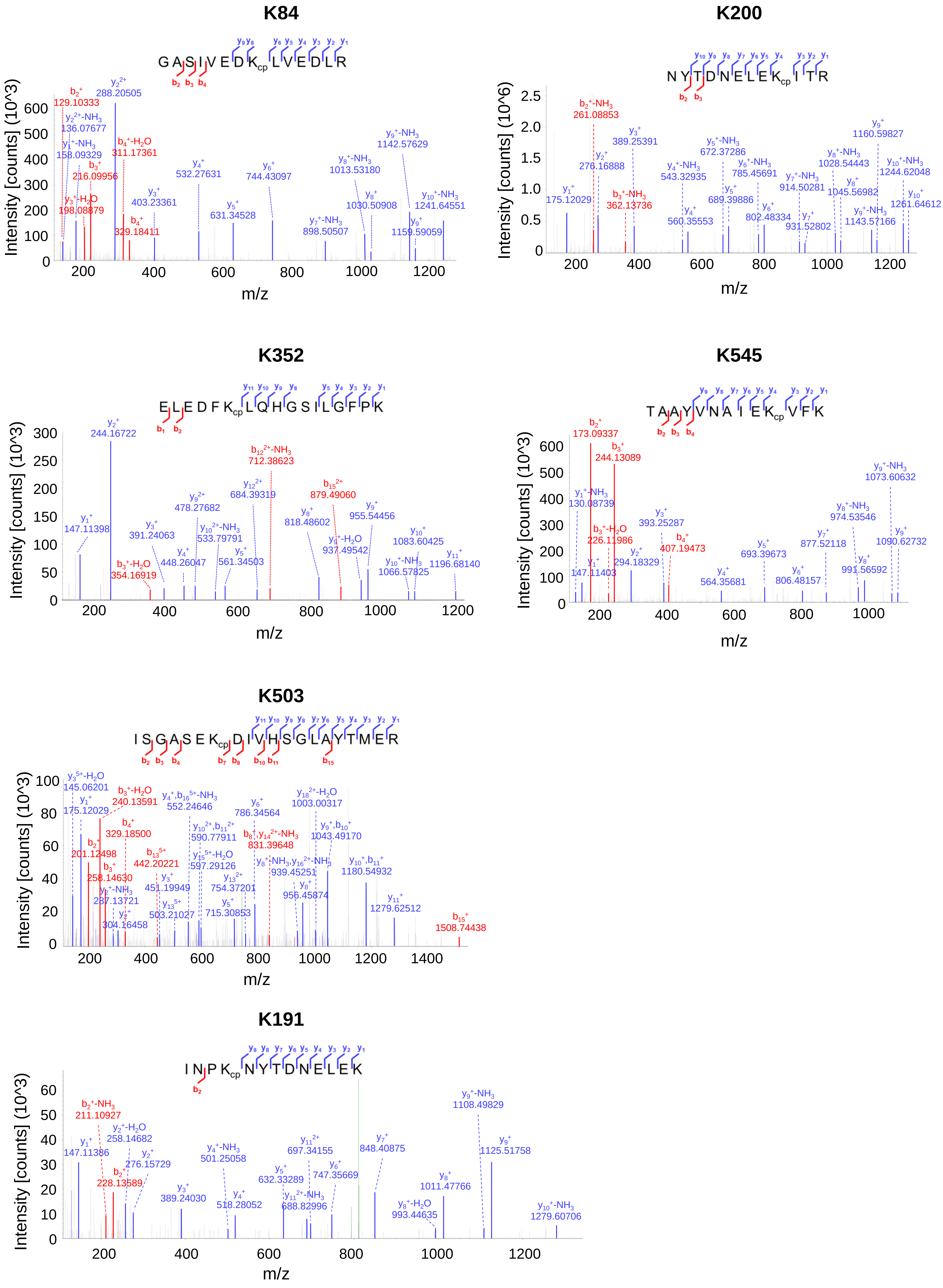
K84
G A S I V E D Kcp L V E D L R
b2+ 129.10333
y2 2+ 288.20505
y2 2+-NH3 136.07677
y1+-NH3 158.09329
b4+-H2O 311.17361
b3+ 216.09956
b3+-H2O 198.08879
b4+ 329.18411
y3+ 403.23361
y4+ 532.27631
y5+ 631.34528
y6+ 744.43097
y7+-NH3 898.50507
y8+-NH3 1013.53180
y8+ 1030.50908
y9+-NH3 1142.57629
y9+ 1159.59059
y10+-NH3 1241.64551
Intensity [counts] (10^3)
m/z
K200
N Y T D N E L E Kcp I T R
b2+-NH3 261.08853
y1+ 175.12029
y2+ 276.16888
y3+ 389.25391
b3+-NH3 362.13736
y4+-NH3 543.32935
y4+ 560.35553
y5+-NH3 672.37286
y5+ 689.39886
y6+-NH3 785.45691
y6+ 802.48334
y7+-NH3 914.50281
y7+ 931.52802
y8+-NH3 1028.54443
y8+ 1045.56982
y9+ 1160.59827
y9+-NH3 1143.57166
y10+-NH3 1244.62048
y10+ 1261.64612
Intensity [counts] (10^6)
m/z
K352
E L E D F Kcp L Q H G S I L G F P K
y2+ 244.16722
y1+ 147.11398
y3+ 391.24063
b3+-H2O 354.16919
y4+ 448.26047
y9 2+ 478.27682
y10 2+-NH3 533.79791
y5+ 561.34503
y12 2+ 684.39319
b12 2+-NH3 712.38623
y8+ 818.48602
b15 2+ 879.49060
y9+ 955.54456
y9+-H2O 937.49542
y10+ 1083.60425
y10+-NH3 1066.57825
y11+ 1196.68140
Intensity [counts] (10^3)
m/z
K545
T A A Y V N A I E Kcp V F K
b2+ 173.09337
b3+ 244.13089
y1+-NH3 130.08739
b3+-H2O 226.11986
y1+ 147.11403
y2+ 294.18329
y3+ 393.25287
b4+ 407.19473
y4+ 564.35681
y5+ 693.39673
y6+ 806.48157
y7+ 877.52118
y8+-NH3 974.53546
y8+ 991.56592
y9+-NH3 1073.60632
y9+ 1090.62732
Intensity [counts] (10^3)
m/z
K503
I S G A S E Kcp D I V H S G L A Y T M E R
y3 5+-H2O 145.06201
y1+ 175.12029
b3+-H2O 240.13591
b4+ 329.18500
b2+ 201.12498
b3+ 258.14630
y2+-NH3 287.13721
y2+ 304.16458
b13 5+ 442.20221
y3+ 451.19949
y4+,b16 5+-NH3 552.24646
y10 2+,b11 2+ 590.77911
y15 5+-H2O 597.29126
y13 5+ 503.21027
y13 2+ 754.37201
y5+ 715.30853
y6+ 786.34564
b8+,y14 2+-NH3 831.39648
y8+-NH3,y16 2+-NH3 939.45251
y8+ 956.45874
y18 2+-H2O 1003.00317
y9+,b10+ 1043.49170
y10+,b11+ 1180.54932
y11+ 1279.62512
b15+ 1508.74438
Intensity [counts] (10^3)
m/z
K191
I N P Kcp N Y T D N E L E K
b2+-NH3 211.10927
y1+ 147.11386
y2+-H2O 258.14682
y2+ 276.15729
b2+ 228.13589
y3+ 389.24030
y4+-NH3 501.25058
y4+ 518.28052
y5+ 632.33289
y11 2+ 697.34155
y11 2+-NH3 688.82996
y6+ 747.35669
y7+ 848.40875
y8+ 1011.47766
y8+-H2O 993.44635
y9+-NH3 1108.49829
y9+ 1125.51758
y10+-NH3 1279.60706
Intensity [counts] (10^3)
m/z

Fig. S6 Carbamylation sites identified in GDH.

MS/MS spectra of the possible carbamylated sites in GDH from tryptic peptide libraries of HEK293T cells.

Fig.S7

a

	162	352	503
Homo sapiens	VSVDEVKALASL	KELEDFKLQHGS	ISGASEKDIVHS
Mus musculus	VSVDEVKALASL	KELEDFKLQHGS	ISGASEKDIVHS
Rattus norvegicus	VSVDEVKALASL	KELEDFKLQHGS	ISGASEKDIVHS
Gallus gallus	VSVDEVKALASL	KELEDYKLQHGT	ISGASEKDIVHS
Drosophila C.	VSVDEVKALASL	KLLEDYKNEHGT	ISGASEKDIVHS
Trachemys scripta elegans	VSRDEVKALASL	KELEDYKLQHGT	ISGASEKDIVHS

b

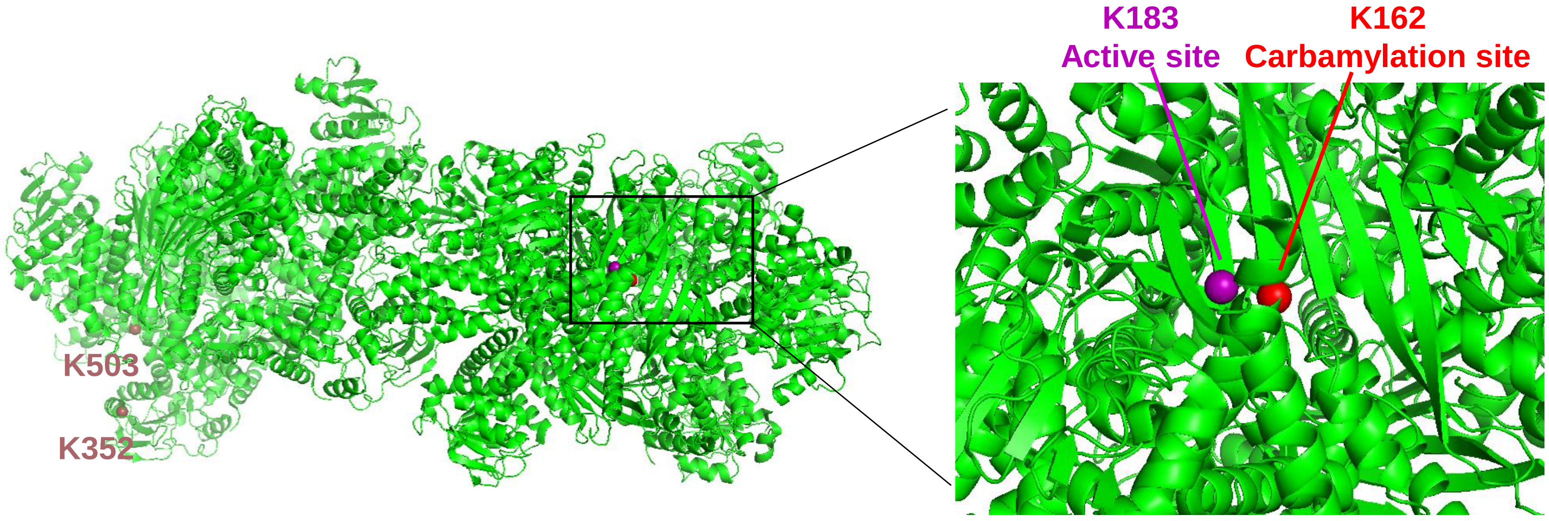

Fig. S7 K162 was a major GDH regulatory carbamylation site.

a K162 is evolutionarily conserved. Alignment of the amino acid sequences of K162, K352, and K503 from *Trachemys scripta elegans* to *Homo sapiens*.

b GDH K162 is adjacent to the catalytically active site. Structural modelling (PyMOL 2.1) revealed that K162 (red) is located near the catalytically active site of GDH K183 (purple).

Fig.S8

a

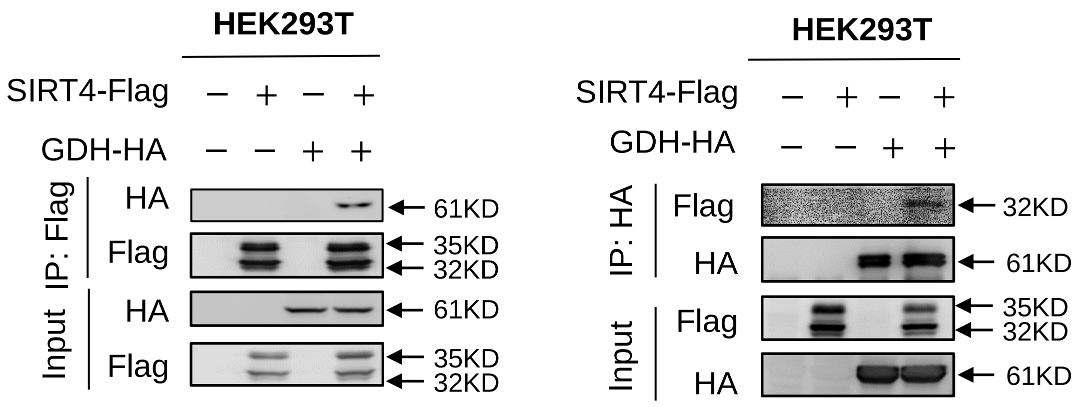
HEK293T
SIRT4-Flag
GDH-HA
IP: Flag
Input
HA
Flag
61KD
35KD
32KD
HEK293T
SIRT4-Flag
GDH-HA
IP: HA
Input
Flag
HA
32KD
61KD
35KD
32KD

b

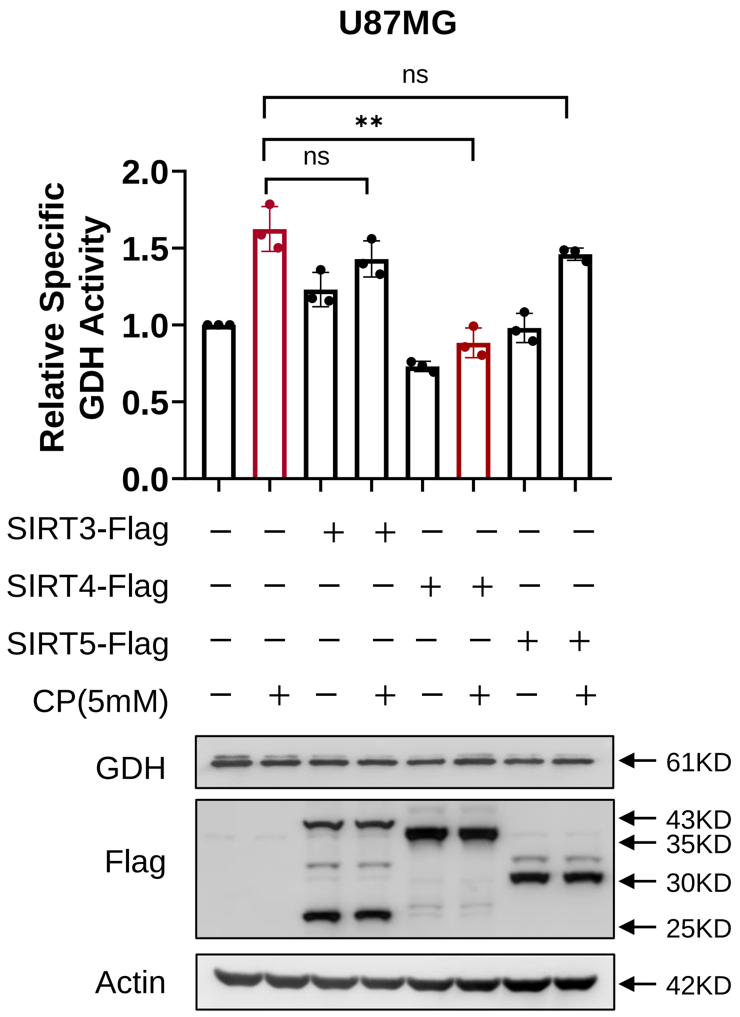
U87MG
ns
**
ns
Relative Specific GDH Activity
2.0
1.5
1.0
0.5
0.0
SIRT3-Flag
SIRT4-Flag
SIRT5-Flag
CP(5mM)
GDH
Flag
Actin
61KD
43KD
35KD
30KD
25KD
42KD

c

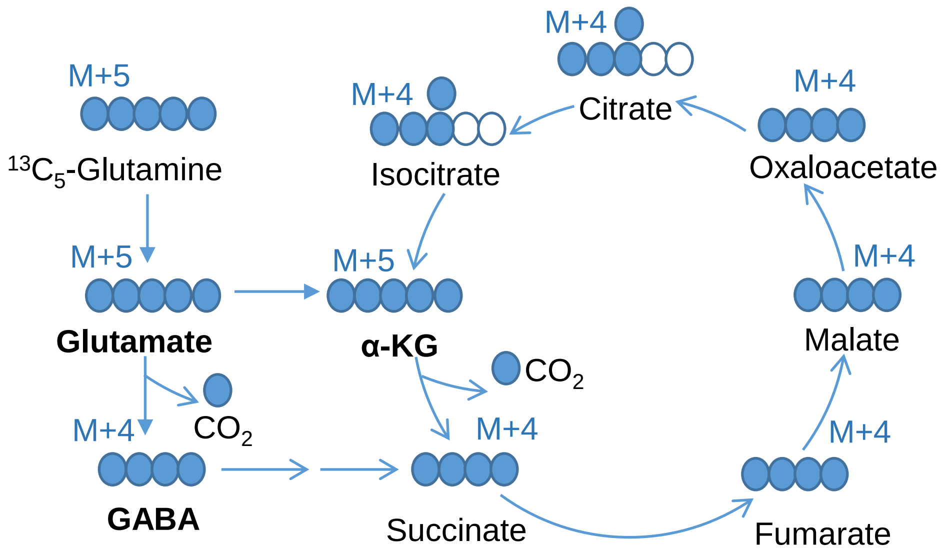
M+5
$^{13}C_5$-Glutamine
M+5
Glutamate
M+4
CO_2
GABA
M+4
Isocitrate
M+4
Citrate
M+4
Oxaloacetate
M+5
α-KG
CO_2
M+4
Succinate
M+4
Malate
M+4
Fumarate

Fig. S8 SIRT4 decarbamylated and inactivated GDH.

a GDH interacted with SIRT4. Flag-tagged SIRT4 was ectopically expressed in HEK293T cells, together with HA-tagged GDH. Proteins were purified via IP with Flag beads, followed by western blot to detect GDH using an HA antibody. Conversely, GDH proteins were purified via IP with Protein A beads conjugated with HA antibody, followed by western blot to detect SIRT4 with Flag antibody.

b SIRT4 decreased GDH carboxylation and endogenous GDH activity in U87MG cells. SIRT3, SIRT4, and SIRT5 were ectopically expressed in U87MG cells along with GDH. The specific activity of GDH in untreated and 5 mM CP-treated cells was determined.

c Schematic diagram of the formation of the M+4 and M+5 forms of labeled metabolites from ^{13}C-glutamine.

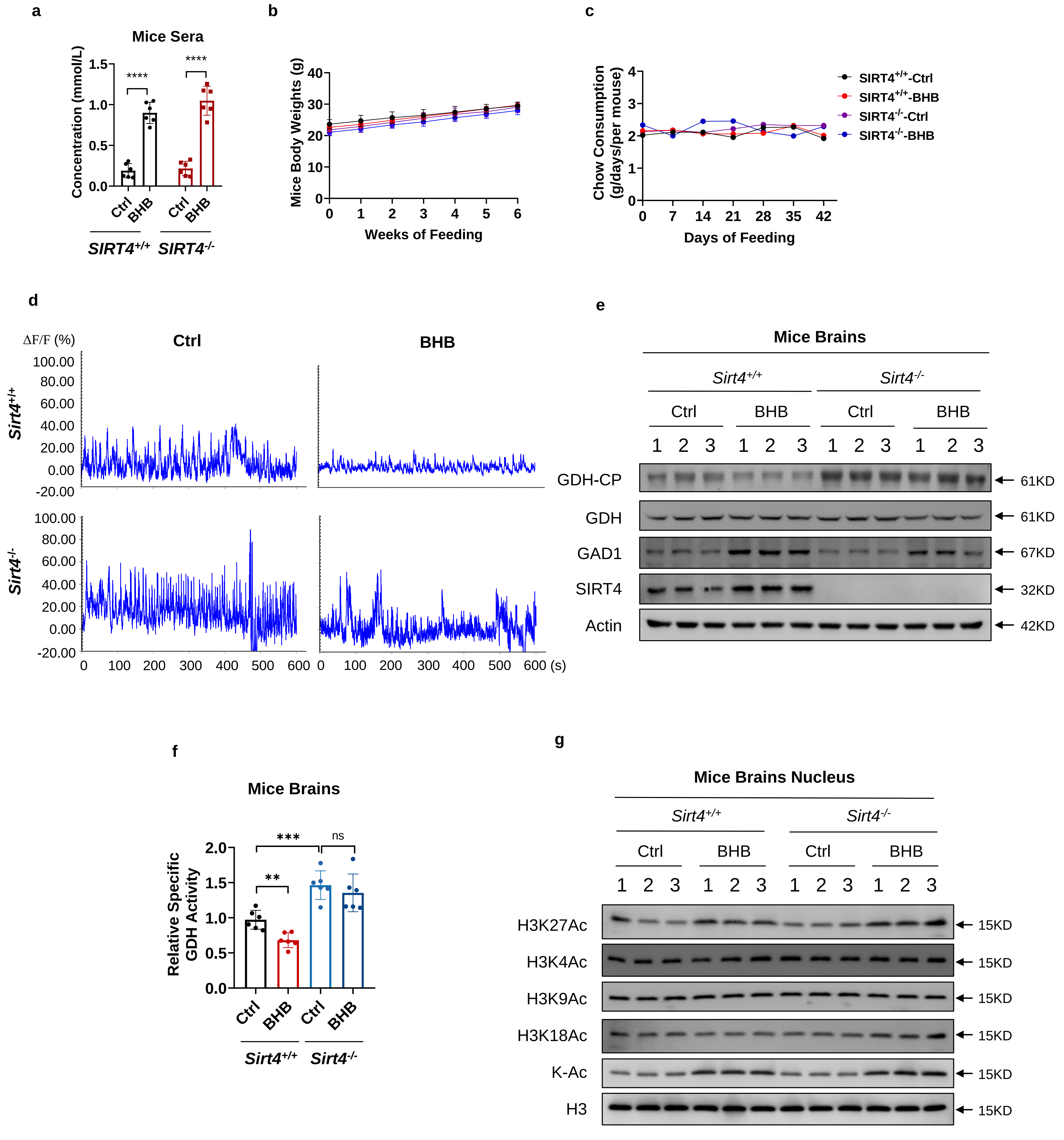
a
Mice Sera
Concentration (mmol/L)
****
****
Ctrl
BHB
SIRT4+/+
SIRT4-/-
b
Mice Body Weights (g)
Weeks of Feeding
c
Chow Consumption (g/days/per mouse)
Days of Feeding
SIRT4+/+-Ctrl
SIRT4+/+-BHB
SIRT4-/-Ctrl
SIRT4-/-BHB
d
ΔF/F (%)
Sirt4+/+
Sirt4-/-
(s)
e
Mice Brains
GDH-CP
GDH
GAD1
SIRT4
Actin
61KD
67KD
32KD
42KD
f
Relative Specific GDH Activity
***
**
ns
g
Mice Brains Nucleus
H3K27Ac
H3K4Ac
H3K9Ac
H3K18Ac
K-Ac
H3
15KD

Fig. S9 BHB was an effective epilepsy inhibitor.

a BHB-chow elevated mice sera BHB. WT and *SIRT4*$^{-/-}$ mice were administered with BHB esters (HVMN), 200 µl by oral gavage daily, 80 mg per mouse, and sera concentrations of BHB were determined after feeding.

b-c Body weights (**b**) and food consumption (**c**) were monitored for C57 and *Sirt4*$^{-/-}$ mice that were fed with or without BHB. Data are mean ± s.d., n = 10 mice in each treatment.

d The ND- and BHB-fed WT and *Sirt4*$^{-/-}$mice were subject to measurements after they were administered PTZ. Video/EEG were monitored. Data are mean ± s.d., n = 10 mice in each treatment.

e BHB increased the protein levels of GAD1 and SIRT4 and the carbamylation levels of GDH in *Sirt4*$^{-/-}$ mouse brains. The GDH protein was purified using Protein A beads conjugated with the GDH antibody from the brains of WT and *Sirt4*$^{-/-}$ mice that were fed BHB. GDH carbamylation was detected for purified GDH.

f GDH was inactivated by the BHB only in WT mouse brains. The WT and *Sirt4*$^{-/-}$ mice were fed with or without BHB. The mouse brain GDH purified at week 20 was assayed for specific activity.

g BHB elevated H3K27Ac in *Sirt4*$^{-/-}$ mouse brains. Histone acetylation levels were assayed in WT and *Sirt4*$^{-/-}$ mouse brains nucleus, employing site-specific and pan-anti-acetyl-lysine (Ac) antibodies.

Fig.S10

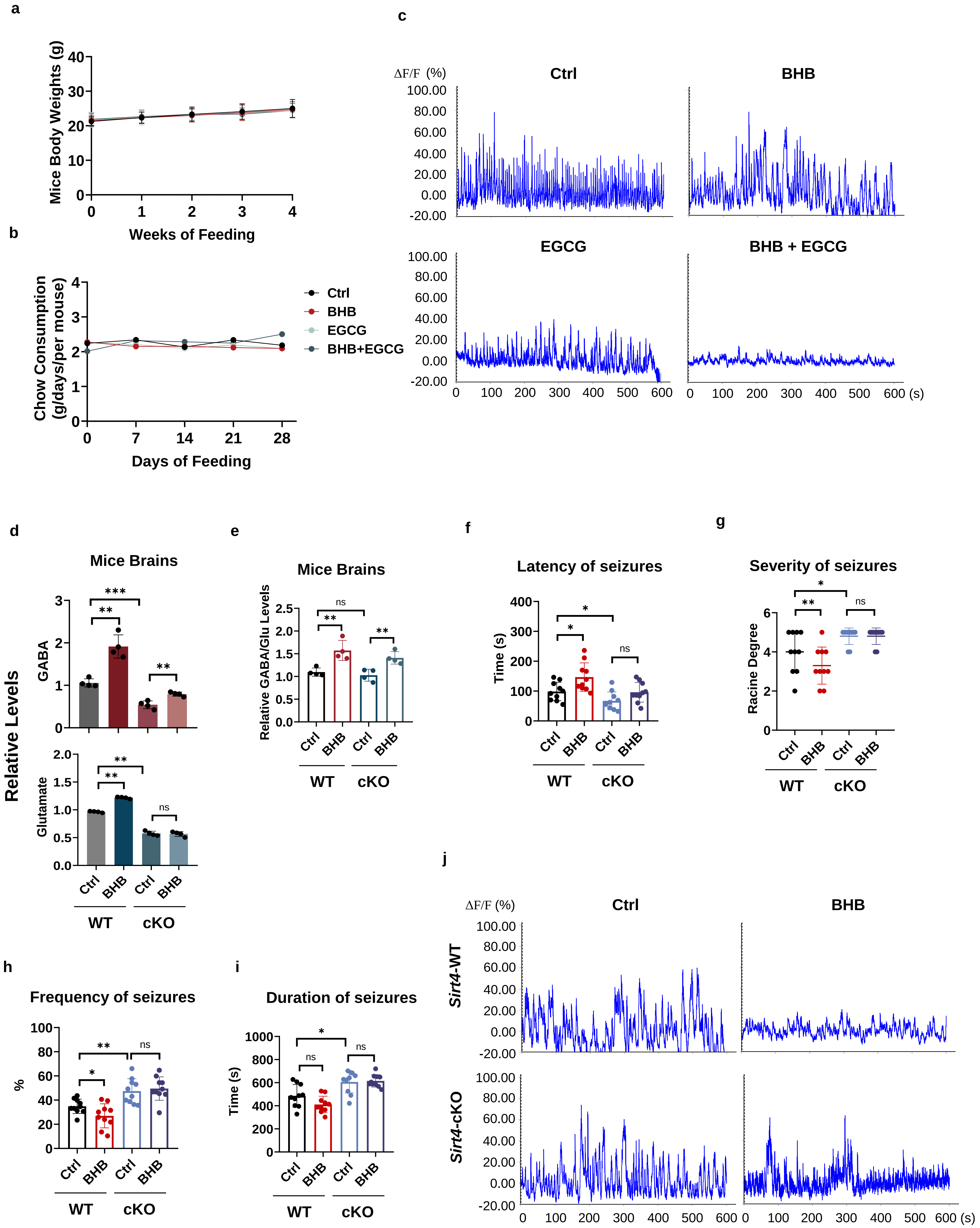
a
Mice Body Weights (g)
Weeks of Feeding
b
Chow Consumption (g/days/per mouse)
Days of Feeding
Ctrl
BHB
EGCG
BHB+EGCG
c
ΔF/F (%)
Ctrl
BHB
EGCG
BHB + EGCG
(s)
d
Mice Brains
Relative Levels
GABA
Glutamate
e
Mice Brains
Relative GABA/Glu Levels
f
Latency of seizures
Time (s)
g
Severity of seizures
Racine Degree
h
Frequency of seizures
%
i
Duration of seizures
Time (s)
j
ΔF/F (%)
Sirt4-WT
Sirt4-cKO
Ctrl
BHB
WT
cKO
ns

Fig. S10 BHB inhibited epilepsy in *Sirt4*-cKO mouse.

a-b BHB and EGCG exerted negligible effects on mice physiologies. Body weights were compared among BHB and/or EGCG treated *Sirt4*$^{-/-}$ mice (**a**), daily food intake was monitored for BHB and/or EGCG treated *Sirt4*$^{-/-}$ mice (**b**). Data were mean ± s.d., n = 10 mice in each treatment.

c BHB or the GDH inhibitor EGCG alone, or BHB together with EGCG were employed to treat *Sirt4*$^{-/-}$ mice. Video/EEG were monitored after mice were administered PTZ.

d-e BHB increased GABA levels (**d**) and the GABA/glutamate ratio (**e**) in WT and *Sirt4*-cKO mouse brains. Data were represented as individual values.

f-j KD SIRT4-dependently relieved seizure phenotypes. The ND- and KD-fed WT and *Sirt4*-cKO mice were subject to measurements after they were administered PTZ. Latency time (**f**), the highest level (**g**), the frequency (**h**), the duration of seizures (**i**) and video/EEG (**j**) were monitored. Data were mean ± s.d., n = 10 mice in each treatment.

KEY RESOURCES TABLE

Reagent or Resource	Resource	Identifier
**Antibodies**		
Anti-SIRT4	Sigma-Aldrich	Cat# HPA029691; RRID: AB_10600118
Anti-GAD1	Proteintech	Cat# 10408-1-AP; RRID: AB_2107733
Anti-Carbamyl-lysine	Abcam	Cat# ab175132;
Anti-GDH	ABclonal	Cat# A5176 ; RRID : AB_2863476
Anti-GOT2	ABclonal	Cat# A6915; RRID: AB_2767474
Anti-GPT	ABclonal	Cat# A2814; RRID: AB_2764652
Anti-GLS	ABclonal	Cat# A3885; RRID: AB_2765362
Anti-GLUL	Cell Signaling Technology	Cat# 80636; RRID: AB_2799956
Anti-BDH1	Santa Cruz	Cat# sc-514413; RRID: AB_2765257
Anti-mono-ADP-ribose binding reagent	Millipore	Cat# MABE1076; RRID: AB_2665469
Anti-HDAC1	Cell Signaling Technology	Cat# 34589; RRID: AB_2756821
Anti-HDAC2	Cell Signaling Technology	Cat# 57156; RRID: AB_2756828
Anti-Histone 3.1	Abmart	Cat# P30266M; RRID: AB_2631273
Anti-Pan-acetyllysine antibody	Home made	N/A
Anti-H3K9Ac	Abcam	Cat# ab177177
Anti-H3K18Ac	Abcam	Cat# ab40888
Anti-H3K27Ac	Abcam	Cat# ab177178
Anti-H3K4Ac	Abcam	Cat# ab176799
Anti-GAPDH	Cell Signaling Technology	Cat# 5174; RRID: AB_10622025
Anti-Flag	Sigma-Aldrich	Cat# F3165; RRID: AB_259529
Anti-HA	Sigma-Aldrich	Cat# H6908; RRID: AB_260070
Anti-Actin	Sigma-Aldrich	Cat# A2066; RRID: AB_476693
anti-Rabbit secondary antibodies	GenScript	Cat#A00098
anti-Mouse secondary antibodies	GenScript	Cat#A00160

**Chemicals, Peptides, and Recombinant Proteins**		
Glutamine-$^{13}C_5$	Sigma-Aldrich	Cat# 605166
NAD^+	Sigma-Aldrich	Cat# V900401
Carbamyl phosphate disodium salt	Sigma-Aldrich	Cat# C4135
Penicillin-Streptomycin	Invitrogen	Cat# 15070063
Pentylenetetrazole	Sigma-Aldrich	Cat# P6500
(-)-Epigallocatechin Gallate (EGCG)	MCE	Cat# HY-13653
(R)-(−)-3-Hydroxybutyric acid sodium salt	Sigma-Aldrich	Cat# 298360
Methyl acetoacetate sodium salt	Sigma-Aldrich	Cat# 00505
NaCl	Sigma-Aldrich	Cat# S9888
Trichostatin A (TSA)	Selleck	Cat# S1045
Romidepsin (FK228)	Selleck	Cat# S3020
TMP195	Selleck	Cat# S8502
RGFP966	Selleck	Cat# S7229
Actinomycin D	Selleck	Cat# S8964
DNase I	Thermo Fisher Scientific	Cat# EN0521
Cycloheximide	MCE	Cat# HY-12320
DTT	Sigma-Aldrich	Cat# 43819
Pyridoxal 5′-phosphate hydrate	Sigma-Aldrich	Cat# P9255
AET	Sigma-Aldrich	Cat# A5879
Sodium glutamate	Sigma-Aldrich	Cat# G5889
**Commercial Assays**		
Glutamate Dehydrogenase Activity Assay Kit	Sigma-Aldrich	Cat# MAK099
Mitochondrial Isolation Kit for Mouse Tissue	Invent Biotechnology	Cat# MM-038
BCA Protein Assay Kit	Beyotime	Cat# P0010S
Ketone Body Assay	Abnova	Cat# KA1630
β-hydroxybutyrate Assay Kit	Sigma-Aldrich	Cat# MAK041
Goat Acetone Elisa Kit	MyBioSource	Cat# MBS9310393
ChIP Assay Kit	Beyotime	Cat# P2078

**Experimental Models: Cell Lines**		
U87MG	Stem Cell Bank, Chinese Academy of Sciences	Cat# TCHu138
Neuro-2a	Stem Cell Bank, Chinese Academy of Sciences	Cat# TCM29
HEK293T	Stem Cell Bank, Chinese Academy of Sciences	Cat# GNHu17
HeLa	Stem Cell Bank, Chinese Academy of Sciences	Cat# TCHu187
**Experimental Models: Organism/Strains**		
Mouse: C57BL/6	Beijing Vital River Laboratory Animal Technology Co., Ltd.	N/A
Mouse: SIRT4 KO (Cas9) C57BL/6	Shanghai GemPharmatech Co., Ltd	N/A
**Recombinant DNA**		
pcDNA3.1-flag-SIRT4	This Work	N/A
pcDNA3.1-flag-GAD1	This Work	N/A
pSUMOH10-$_{27\text{-}314}$SIRT4	This Work	N/A
pcDNA3.1-HA-SIRT3		
pcDNA3.1-HA-SIRT4	This Work	N/A
pcDNA3.1-HA-SIRT5	This Work	N/A
pcDNA3.1-flag-GLUD1	This Work	N/A
pcDNA3.1-flag-GLUD1^{C119G}	This Work	N/A
pcDNA3.1-flag-GLUD1^{K84R}	This Work	N/A
pcDNA3.1-flag-GLUD1^{K162R}	This Work	N/A
pcDNA3.1-flag-GLUD1^{K191R}	This Work	N/A
pcDNA3.1-flag-GLUD1^{K200R}	This Work	N/A
pcDNA3.1-flag-GLUD1^{K352R}	This Work	N/A
pcDNA3.1-flag-GLUD1^{K503R}	This Work	N/A
pcDNA3.1-HA-BDH1	This Work	N/A
pcDNA3.1-HA-HDAC1	This Work	N/A
pcDNA3.1-HA-HDAC2	This Work	N/A

pcDNA3.1-flag-CPS1	This Work	N/A
**Primers**		
si*SIRT4*-F	ACUCAUUGGUUCCUUUAAGGG	
si*SIRT4*-R	CUUAAAGGAACCAAUGAGUCC	
si*BDH1*-F	AAGUAGAACCAAGCAAUAGUG	
si*BDH1*-R	CUAUUGCUUGGUUCUACUUCC	
si*GLUD1*-F	UUCAUUAUCAGUAUAGUUCUU	
si*GLUD1*-R	GAACUAUACUGAUAAUGAAUU	
sg*HDAC1*-F	caccgCACCATTCGTAACGTTGCC	
sg*HDAC1*-R	aaacGGCAACGTTACGAATGGTGc	
sg*HDAC2*-F	caccgCCGTAATGTTGCTCGATGT	
sg*HDAC2*-R	aaacACATCGAGCAACATTACGGc	
Sirt4-KO-F1	ATCAAAAGCTGCACCACCACTG	
Sirt4-KO-R1	TCTGGTGTTCTGTCTTGACACGC	
Sirt4-KO-F2	TTGCAGACATACCGGAAGAAGG	
Sirt4-KO-R2	TTCAGGCAAGCCAAATCGTCAG	
**Software and Algorithms**		
Graphpad Prism 8.0	Graphpad Software	https://www.graphpad.com
Image J	NIH	https://imagej.nih.gov/ij/
PyMOL 2.1	Schrodinger	https://pymol.org/2/
